# Practice Guidelines for Ocular Telehealth-Diabetic Retinopathy, Third Edition

**DOI:** 10.1089/tmj.2020.0006

**Published:** 2020-04-16

**Authors:** Mark B. Horton, Christopher J. Brady, Jerry Cavallerano, Michael Abramoff, Gail Barker, Michael F. Chiang, Charlene H. Crockett, Seema Garg, Peter Karth, Yao Liu, Clark D. Newman, Siddarth Rathi, Veeral Sheth, Paolo Silva, Kristen Stebbins, Ingrid Zimmer-Galler

**Affiliations:** ^ 1^ Indian Health Service-Joslin Vision Network (IHS-JVN) Teleophthalmology Program, Phoenix Indian Medical Center, Phoenix, Arizona.; ^ 2^ Division of Ophthalmology, Department of Surgery, Larner College of Medicine, University of Vermont, Burlington, Vermont.; ^ 3^ Beetham Eye Institute, Joslin Diabetes Center, Massachusetts.; ^4^Department of Ophthalmology, Harvard Medical School, Boston, Massachusetts.; ^5^ Department of Ophthalmology and Visual Sciences, The University of Iowa, Iowa City, Iowa.; ^6^ Department of Biomedical Engineering, and The University of Iowa, Iowa City, Iowa.; ^7^ Department of Electrical and Computer Engineering, The University of Iowa, Iowa City, Iowa.; ^8^Department of Ophthalmology, Stephen A. Wynn Institute for Vision Research, The University of Iowa, Iowa City, Iowa.; ^9^Iowa City VA Health Care System, Iowa City, Iowa.; ^10^ IDx, Coralville, Iowa.; ^11^ Arizona Telemedicine Program, The University of Arizona, Phoenix, Arizona.; ^12^ Department of Ophthalmology, Casey Eye Institute, Oregon Health and Science University, Portland, Oregon.; ^13^ Department of Medical Informatics and Clinical Epidemiology, Oregon Health and Science University, Portland, Oregon.; ^14^ Department of Ophthalmology, Baylor College of Medicine City, Houston, Texas.; ^15^ Department of Ophthalmology, University of North Carolina, Chapel Hill, North Carolina.; ^16^ Oregon Eye Consultants, Eugene, Oregon.; ^17^ Department of Ophthalmology and Visual Sciences, University of Wisconsin-Madison, Madison, Wisconsin.; ^18^ Plaza Vision Center, Dallas, Texas.; ^19^ Department of Ophthalmology, NYU Langone Health, New York, New York.; ^20^ University Retina and Macula Associates, University of Illinois at Chicago, Chicago, Illinois.; ^21^ Vision Care Department, Hillrom, Skaneateles Falls, New York, New York.; ^22^ Wilmer Eye Institute, Johns Hopkins University, Baltimore, Maryland.

**Keywords:** teleophthalmology, telemedicine, telehealth, diabetic retinopathy, artificial intelligence

## Abstract

The following document and appendices represent the third edition of the *Practice Guidelines for Ocular Telehealth-Diabetic Retinopathy*. These guidelines were developed by the Diabetic Retinopathy Telehealth Practice Guidelines Working Group. This working group consisted of a large number of subject matter experts in clinical applications for telehealth in ophthalmology.

The editorial committee consisted of Mark B. Horton, OD, MD, who served as working group chair and Christopher J. Brady, MD, MHS, and Jerry Cavallerano, OD, PhD, who served as cochairs. The writing committees were separated into seven different categories. They are as follows:

1. Clinical/operational: Jerry Cavallerano, OD, PhD (Chair), Gail Barker, PhD, MBA, Christopher J. Brady, MD, MHS, Yao Liu, MD, MS, Siddarth Rathi, MD, MBA, Veeral Sheth, MD, MBA, Paolo Silva, MD, and Ingrid Zimmer-Galler, MD.
2. Equipment: Veeral Sheth, MD (Chair), Mark B. Horton, OD, MD, Siddarth Rathi, MD, MBA, Paolo Silva, MD, and Kristen Stebbins, MSPH.
3. Quality assurance: Mark B. Horton, OD, MD (Chair), Seema Garg, MD, PhD, Yao Liu, MD, MS, and Ingrid Zimmer-Galler, MD.
4. Glaucoma: Yao Liu, MD, MS (Chair) and Siddarth Rathi, MD, MBA.
5. Retinopathy of prematurity: Christopher J. Brady, MD, MHS (Chair) and Ingrid Zimmer-Galler, MD.
6. Age-related macular degeneration: Christopher J. Brady, MD, MHS (Chair) and Ingrid Zimmer-Galler, MD.
7. Autonomous and computer assisted detection, classification and diagnosis of diabetic retinopathy: Michael Abramoff, MD, PhD (Chair), Michael F. Chiang, MD, and Paolo Silva, MD.

## Table of Contents

**CONTRIBUTORS**                    495
**PREAMBLE**                    496
**SCOPE**                    497
**INTRODUCTION**                    497
**PRACTICE GUIDELINES**                    498
**Clinical guidelines**                    498
  I. Principles of an ocular telehealth program for DR                    498
    A. Mission                    498
    B. Vision                    498
    C. Goals                    498
    D. Guiding principles                    498
  II. Ethics                    498
  III. Clinical validation                    498
    A. Category 1                    499
    B. Category 2                    499
    C. Category 3                    500
    D. Category 4                    500
  IV. Communication                    501
  V. Personnel qualifications                    501
    A. Medical care supervisor                    501
    B. Patient care coordinator                    501
    C. Image acquisition personnel                    501
    D. Image review and evaluation personnel                    501
    E. Information systems personnel                    501
**Technical guidelines**                    501
  I. Equipment specifications                    501
    A. Image acquisition                    503
    B. Image display                    503
    C. Image analysis                    504
  II. Data management                    504
    A. Interoperability                    504
    B. Compression                    504
    C. Data communication and transmission                    504
    D. Archiving and retrieval                    504
    E. Security                    505
    F. Reliability and redundancy                    505
    G. Documentation                    505
**Administrative guidelines**                    505
  I. Legal requirements                    505
    A. Facility accreditation                    505
    B. Health Insurance Portability and Accountability Act                    505
    C. Privileging and credentialing                    505
    D. Fraud and abuse                    505
    E. State medical practice acts/licensure                    506
    F. Tort liability                    506
    G. Consent                    507
  II. Quality control                    507
  III. Operations                    507
  IV. Customer support                    507
    A. Originating site                    507
    B. Data transmission                    508
    C. Distant site                    508
  V. Financial factors                    508
    A. Reimbursement                    508
    B. Grants                    508
    C. Federal programs                    508
    D. Other financial factors                    508
    E. Equipment cost                    509
**TABLES AND FIGURES**                    499,502,503,512,514,520,522
**ABBREVIATIONS**                    509
**GLOSSARY**                    509
**APPENDICES**                    510
  1. Clinical Validation                    510
  2. Interoperability                    511
  3. Autonomous and Computer-Assisted Detection, Classification, and Diagnosis of Diabetic Retinopathy                    513
  4. Health Insurance Portability and Accountability Act                    519
  5. Privileging and Credentialing                    519
  6. Quality Control                    520
  7. Customer Support                    521
  8. Reimbursement                    522
  9. Telemedicine for Glaucoma: Guidelines and Recommendations                    524
  10. Telemedicine for Retinopathy of Prematurity                    527
  11. Telemedicine for Age-Related Macular Degeneration                    533
**REFERENCES**                    535

## Preamble

The American Telemedicine Association (ATA) brings together diverse groups from traditional medicine, academia, technology, telecommunications companies, e-health, allied professional and nursing associations, medical associations, government, military, regulatory, and other stakeholders to address and advance compliance with legal, ethical, and professional standards in the practice of telemedicine. The ATA has embarked on an organized effort to establish guidelines for the practice of telemedicine in various clinical applications to define patient and provider expectations, aspire to uniform quality of service for patients and providers, enhance patient experience, and enable providers to deliver appropriate care using evidence-based practices.

The guidelines are developed by panels that include experts from the field and other strategic stakeholders, and are designed to serve as a standard reference and educational tool for professionals using telehealth tools for health care service delivery. The process for developing these guidelines is based on evidence, professional consensus, and a rigorous review, including open public commentary period, with final approval by the ATA Board of Directors. Guidelines are reviewed and updated periodically.

The purpose of these guidelines is to assist providers in pursuing a sound course of action in providing safe and effective medical care using telehealth tools based on current scientific knowledge, technological requirements, and patient needs. Safe and effective practice requires technical training, professional knowledge and skill, and explicit processes as described in each document.

Adherence to these guidelines alone will not guarantee accurate diagnoses, appropriate clinical treatment, or optimal outcomes. Appropriate divergence from the guidelines may be indicated under certain conditions, such as emergency situations or locations with limited resources or other unavoidable constraints. Similarly, technological advances may alter prevailing practices or provide new and expanded opportunities.

The guidelines in this document are based on the accumulated knowledge and experience of the ATA workgroups, eye care and telemedicine professionals, and other stakeholders, and generally describe the evidenced-based best practices for ocular telehealth. However, the technical and administrative guidelines do not purport to establish binding legal standards for delivering telemedicine services.

The previous ATA Ocular Telehealth Diabetic Retinopathy Practice Guidelines were issued in 2011. This third edition reflects new evidence, new technologies, and expanded scope of the ocular telehealth domain. All guidelines issued by the ATA are properties of the ATA. Any modification or reproduction of the published guidelines must receive prior approval by the ATA.

## Scope

The following document includes fundamental requirements to be followed when providing medical and other health care services using telecommunication technologies, and any other electronic communications between patients, practitioners, and other health care providers, as well as “best practice” recommendations. The guidelines apply to individual practitioners, group and specialty practices, hospitals and health care systems, and other providers of health-related services where there are telehealth interactions between patients and health care service providers.

When guidelines, position statements, or standards from any other professional organization or society exist, health professionals should also review these documents and, as appropriate, incorporate them into practice.

These guidelines pertain primarily to health care professionals and patients located in the United States. In situations wherein either or both parties are not within the United States, these guidelines may be referenced, but any local guidelines that are in place should take precedence.^1–3^

These guidelines are intended to be used as a companion to the *ATA Core Operational Guidelines for Telehealth Services*.^4^ Recommendations in the core guidelines are not repeated herein except to emphasize or expand upon a particular point, or to provide domain-specific detail. The reader **should** review the core guidelines first to provide the context for proper understanding and implementation of the *Practice Guidelines for Ocular Telehealth-Diabetic Retinopathy*.

The guidelines address three aspects of service delivery: clinical, technical, and administrative. Based upon the quantity and quality of peer-reviewed evidence, the guidelines are classified into four levels of adherence:

- “**Shall**” indicates required action whenever feasible and/or practical.
- “**Shall not**” indicates a proscription or action that is strongly advised against.
- “**Should**” indicates a recommended action without excluding others.
- “**May**” indicates pertinent actions that may be considered to optimize the telemedicine encounter or operational process.

These indications are found in bold throughout the document.

## Introduction

These guidelines present recommendations for designing, implementing, and operating an ocular telehealth diabetic retinopathy (DR) program in a broad range of clinical settings and targeted outcomes. This document also addresses current clinical, technical, and administrative issues that form the basis for evaluating DR telehealth techniques and technologies. These guidelines are intended to be consistent with federal regulations and industry best practices at the time of publication that emphasize clinical quality, data security and integrity, and interoperable health information exchange. Federal, state, and regional regulations supersede the recommendations in these guidelines. This document will be reviewed periodically and revised to reflect evolving technologies, evidence, regulations, and clinical guidelines.

This third edition of the guidelines includes four new clinical appendices that introduce additional ocular telehealth domains (*Appendices A3, A9–A11*). These are planned for future development into independent guidelines to be included in an ocular telehealth suite of practice guidelines.

## Practice Guidelines

### Clinical Guidelines

#### I. Principles of an ocular telehealth program for DR

Private individuals, public and private organizations, and national and international agencies may undertake telemedicine programs for DR that have been shown to be efficacious, cost-effective, and scalable means to identify diabetes-related eye disease and thereby prevent visual loss. Designing, building, implementing, and sustaining an ocular telehealth DR program require clearly defined mission, vision, goals, and guiding principles. The following statements are a guide for leadership and staff in developing and sustaining appropriate and effective programs.

##### A. Mission

Increase cost-effective and culturally sensitive access and adherence to accepted standards of eye care for people with diabetes mellitus (DM).

##### B. Vision

Ocular telehealth can be an integral component of primary care for people with DM by expanding patient-centric access to retinal examinations consistent with evidence-based recommendations for eye care in diabetes.

##### C. Goals

- Improve access to diagnosis and evidence-based management of DR.
- Reduce the incidence of vision loss due to DR.
- Decrease the cost of identifying patients with DR.
- Promote telehealth to enhance the efficiency and clinical effectiveness of evaluation, diagnosis and management of DR.
- Promote telehealth to enhance the availability, patient centricity, quality, efficiency, and cost-effectiveness of remote evaluation and management of DR.
- Facilitate integration of diabetes eye care with primary and specialty medical care.
- Promote widespread adoption of telehealth services for DR.^5^

##### D. Guiding principles

Although ocular telehealth programs offer new opportunities to improve access and quality of care for people with DR, programs **shall** be developed for deployment in a safe and effective manner. Program outcomes **shall** be closely monitored to meet or exceed current standards-of-care for retinal examination and identify opportunities to improve service delivery and clinical outcomes.

DM adversely affects the entire eye and has a diverse influence on visual function. Patients should be aware that a validated teleophthalmology examination of the retina may substitute for a traditional onsite dilated retinal evaluation for DR, but patients **shall** be informed that the examination is not a replacement for a comprehensive eye examination, and does not replace the need for ongoing care by conventional eye examinations.

#### II. Ethics

Regardless of the program, the care of the patient **shall not** be compromised. Telemedicine practice **shall** conform to the same professional ethics that govern in-person care. This responsibility encompasses a broad range of issues including, but not limited to, confidentiality, image quality, data integrity, clinical accuracy, reliability, and adherence to all applicable national and local regulations such as Health Insurance Portability and Accountability Act (HIPAA). Telemedicine programs and providers **shall** incorporate ethical statements and policies and legal/regulatory requirements into their standard operating procedures, including:

- An explicit code of ethics.
- Compliance with applicable federal, state, and jurisdictional laws and regulations, and institutional policies.
- Nondiscrimination clause regarding denial of service to people on the basis of disability, gender, gender preference or sexual orientation, ethnicity, national origin, or religious affiliation.
- Provision of care in a culturally sensitive manner.
- Provision of service not conditional upon receipt of payment by the patient.

#### III. Clinical validation

Multicenter national clinical trials provide evidence-based criteria for clinical guidelines in diagnosing and treating DR (*Appendix A1*). Telehealth programs for DR **shall** define program goals and performance in relation to broadly accepted clinical standards.

Early Treatment Diabetic Retinopathy Study (ETDRS) 30°, stereoscopic, seven-standard field, color, 35 mm slides (based on the ETDRS extension of the modified Airlie House classification of DR,^6^ ETDRS photographs) have been the gold standard for evaluating DR in major clinical trials of DR. Although no standard criteria have been widely accepted as performance measurements of digital imagery used for DR evaluation, current clinical trials sponsored by the National Eye Institute have transitioned to digital images for DR assessment.^7,8^

Telehealth programs for DR **should** demonstrate an ability to compare favorably with ETDRS film or digital photography as reflected in kappa values for agreement of diagnosis, false-positive and false-negative readings, positive predictive value, negative predictive value, sensitivity and specificity of diagnosing levels of DR and diabetic macular edema (DME).^9–12^ Because programs have reported referral-warranted ocular disease in many patients with ungradable images, inability to obtain or grade images **should** be considered a positive finding and patients with unobtainable or ungradable images **should** be promptly reimaged or referred for a more advanced evaluation.^13^

It is recognized that severity levels of DR other than those defined by the ETDRS are used clinically for grading DR (see *Table 1* for comparisons between ETDRS levels of DR and the International Clinical Diabetic Retinopathy Disease Severity Scale, and *Table 2* for comparisons between ETDRS DME and the International Clinical Diabetic Retinopathy Disease Severity Scale).^14^ Protocols **should** state the reference standard used for validation and relevant data sets used for comparison.

**Table 1.** International Clinical Diabetic Retinopathy Scale Compared with Early Treatment Diabetic Retinopathy Study Levels of Diabetic Retinopathy

INTERNATIONAL CLASSIFICATION LEVEL OF DR	ETDRS LEVEL OF DR (ETDRS REPORT 12)
No apparent retinopathy	Level 10; DR absent
	Levels 14, 15; DR questionable
Mild NPDR	Level 20; microaneurysms only
Moderate NPDR	Level 35; mild NPDR
	Levels 43, 47; moderate NPDR
Severe NPDR	Levels 53A–53E; severe NPDR, very severe NPDR
PDR	Level 61; mild PDR
	Level 65; moderate PDR
	Levels 71, 75; high-risk PDR
	Levels 81, 85; advanced PDR

DR, diabetic retinopathy; ETDRS, Early Treatment Diabetic Retinopathy Study; NPDR, nonproliferative diabetic retinopathy; PDR, proliferative diabetic retinopathy.

**Table 2.** International Clinical Diabetic Macular Edema Scale Compared with Early Treatment Diabetic Retinopathy Study Where Noted

DISEASE SEVERITY LEVEL	FINDINGS	DME SCALE
DME apparently absent	No apparent retinal thickening or HE in posterior pole	
DME apparently present	Some apparent retinal thickening or HE in posterior pole	Mild DME: some retinal thickening or HE in posterior pole but distant from center of the macula (ETDRS: DME but not CSME) (not central-involved DME)
		Moderate DME: retinal thickening or HE approaching the center but not involving the center (ETDRS: CSME) (not central-involved DME)
		Severe DME: retinal thickening or HE involving the center of the macula (ETDRS: CSME) (central-involved DME)

CSME, clinically significant macular edema; DME, diabetic macular edema; HE, hard exudates.

This *Practice Guidelines for Ocular Telehealth-Diabetic Retinopathy* defines four major categories of validation for DR telehealth programs using ETDRS photographs as the reference standard. The validation study **shall** be structured to assess the program's “end-to-end” performance rather than any single piece of its technology, and the study design **should** follow conventional scientific methodology. Although ETDRS photographs currently provide an ideal standard for validation, clinical comparators **may** be used for program validation if the examination is conducted by a retinal specialist using accepted best practices.

Validation categories are not a quality continuum, but rather performance categories that describe distinct clinical outcomes of public health relevance reflecting program goals. In addition, they provide a standardized language for communicating performance for clinical, research, reimbursement, request for proposal (RFP), and regulatory compliance purposes. Information about the program's validation study design and performance **should** be publicly available to users and other stakeholders.

##### A. Category 1

Category 1 validation indicates a system can separate patients into one of two groups: (1) those who have no or very mild nonproliferative diabetic retinopathy (NPDR) (ETDRS level 20 or below) and (2) those with levels of mild NPDR or greater (greater than or equal to ETDRS level 35). Functionally, category 1 validation allows screening for presence versus absence of DR.

##### B. Category 2

Category 2 validation indicates a program accurately determines if sight-threatening diabetic retinopathy (STDR) or potentially STDR is present or not present as evidenced by any level of DME, severe or worse levels of NPDR (ETDRS level 53 or worse), or proliferative diabetic retinopathy (PDR) (ETDRS level 61 or worse). Functionally, category 2 allows screening for presence versus absence of STDR or potentially STDR.

##### C. Category 3

Category 3 validation indicates that a program accurately identifies ETDRS-defined clinical levels of NPDR (mild, moderate, or severe), PDR (early and high risk), and DME (central-involved DME or not central-involved DME). Functionally, category 3 validation provides a clinical diagnosis of DR/DME severity to match conventional clinical retinal examination through dilated pupils or ETDRS photographs, allowing remote management of the patient.

##### D. Category 4

Category 4 validation indicates that a program accurately identifies the presence and degree of specific lesions of DR to match the ability of ETDRS photographs to determine all specific lesions and levels of DR and DME, ranging from levels 10 to 90. Functionally, category 4 validation indicates a program can replace or coexist with ETDRS photographs as a gold standard and **may** be used in any clinical or research program.^15^

The validation categories entail all components of a program (end-to-end) and do not refer to any single element such as the retinal imaging device, imaging protocol, image manager, compression protocol, image display, and image review protocol.

Determination of the validation category **should** be done by a properly designed study using ETDRS photographs as controls, although clinical comparators by a retinal specialist **may** be used. The study groups **shall** include statistically appropriate representation from the full range of DR and DME severity from no clinically evident DR/DME to PDR and central-involved DME or not central-involved DME.

Threshold sensitivity and specificity for validation categories 1 and 2 **shall** be 80% and 95%,^16^ respectively, and **shall** be calculated including ungradable images. For categories 3 and 4, a test of categorical agreement such as the kappa statistic with substantial agreement **should** be used. For example, the system of Landis and Koch defines slight agreement as kappa of 0–0.20; fair agreement, 0.21–0.40; moderate agreement, 0.41–0.60; substantial agreement as 0.61–0.80; and almost perfect agreement as >0.81.^11,17^ The threshold for image gradability **shall** be defined in a structured manner, and ungradable images shall be included as a positive finding in statistical analyses.

The study that establishes the program's clinical performance and validation category applies to all its implementations. Clinical fidelity with the validation study is maintained by standardized implementation and ongoing quality assurance (QA) (*Appendix A6*). Accordingly, individual implementations within the original program need not be restudied. However, substantial changes in technology or clinical operations **may** warrant repeat study to re-establish clinical performance and validation category.

A telehealth program's validation category impacts clinical, business, and operational features. The category influences hardware and software technology, staffing and support, clinical workflow and outcomes, participant licensure, QA, and business plan. Equipment cost, technical difficulty, operational complexity, and training requirements increase with increasing program performance as measured by validation category.^18^

A telehealth program's goals and desired performance **may** influence choice of technology and protocol. Some programs use pharmacologic pupil dilation on all or selected patients, whereas others perform imaging with nonmydriatic cameras and undilated pupils. A higher rate of ungradable photographs has been reported through undilated versus dilated pupils.^19–21^ People with diabetes, particularly those >50 years of age, often have smaller pupils and a greater incidence of cataracts, which may limit image quality if performed through an undilated pupil.^22,23^

Pupil dilation is associated with a small risk of angle-closure glaucoma. Although the risk of inducing angle-closure glaucoma with dilation using 0.5% tropicamide is minimal with no reported cases in a large meta-analysis,^24,25^ programs using pupil dilation **shall** have a defined protocol to recognize and address this potential complication. Pupil dilation is not an operational requirement for any particular validation category, but ocular telehealth programs for DR **may** use pupil dilation based upon regulatory dependencies, program preferences, and outcome goals.

Depending on the telehealth program operational preferences and validation category, images **may** be acquired and reviewed stereoscopically. Evidence suggests that accurate identification of macular edema presence or severity may not always be possible using nonstereoscopic modalities.^26^ Without direct assessment of retinal thickening through stereoscopic evaluation or optical coherence tomography (OCT), determination of DME relies upon surrogate lesions of hard exudates or microaneurysms in the macular field.^27,28^ However, macular edema is not completely defined or identifiable by these surrogate markers in all cases.^29,30^ Central-involved DME or not central-involved DME is often accompanied by other DR lesions that may also independently trigger referral.

It is possible that a program without stereoscopic capabilities or OCT may be validated to identify macular edema with acceptable sensitivity,^28,31^ even though stereoscopic evaluation of DME is significantly more sensitive and specific than monoscopic techniques.^21^ Artificial intelligence (AI) algorithms may offer another indirect measure of DME that has sufficient accuracy to warrant clinical applications in some settings^32^ (*Appendix A3*). A program **may** use nonstereoscopic techniques to establish DME severity based upon its operational preferences and demonstrated validation category.

#### IV. Communication

Communication is the foundation of ocular telehealth.^33,34^ Communication **shall** be coordinated and reliable among originating site (OS) and distant site (DS), telehealth providers and patients, and telehealth providers and other members of the patient's health care team. Communication with patients **shall** be aligned with the patients' cultural and physical needs. Providers interpreting retinal telehealth images **shall** render reports in accordance with relevant jurisdictions, community standards, and regulatory requirements. Although reporting is typically provided to referring providers, the program should emphasize consistent and timely communication to the patient of the telemedicine examination outcome.

#### V. Personnel qualifications

Telehealth programs for DR depend upon a variety of functions. Distinct individuals may assume these responsibilities or a person may assume several roles depending on the size and scope of the program. Qualifications of these personnel shall be documented, including initial and recurrent training.

##### A. Medical care supervisor

An appropriately licensed ophthalmologist or optometrist with expertise in evaluation and management of DR **shall** assume ultimate responsibility for the program and is responsible for oversight of image interpretation, report recommendations, and patient safety. Responsibilities include delivering timely recommendations for appropriate care management and providing feedback to the imagers, graders, and other program participants. Responsibilities also include ensuring that all components of the program, including image acquisition, grading, and reporting, are of appropriate quality and that related patient health data meet accepted and expected standards. Nonmedical oversight **may** be used depending on validation category, goals of the program, regulatory requirements, and QA safeguards. This role may include coordinating and tailoring the integration of the telehealth workflow for DR into the local clinical setting.^35^

##### B. Patient care coordinator

The patient care coordinator ensures that each patient receives DR education and completes appropriate follow-up, especially for those meeting criteria for referral. A program **may** use a dedicated position for this role or use a shared position depending on the program size and geographic scope.

##### C. Image acquisition personnel

Image acquisition personnel (“imagers”) are responsible for acquiring retinal images. A licensed eye care professional may not be physically available at all times during a telehealth session, so imagers **shall** possess the knowledge and skills for imaging independently or with assistance and consultation by telephone, including:

- Understanding of basic ocular telehealth technology and principles.
- Qualifications for obtaining appropriate image fields of diagnostic quality.
- Understanding of the clinical appearance of common retinal diseases requiring immediate or urgent evaluation.
- Communication skills for acquiring patient informed consent and providing patient education.
- Basic understanding of angle-closure glaucoma if pupil dilation is performed, including entry-level skills in screening for shallow anterior chamber and recognition of angle-closure signs and symptoms.

##### D. Image review and evaluation personnel

Image review and evaluation specialists (readers) are responsible for timely grading of images for retinal lesions and determining levels of DR. Only qualified readers **shall** perform retinal image grading and interpretation. Qualifications **shall** include academic and clinical training. If a reader is not an optometrist or ophthalmologist, specific training and demonstrated proficiency **shall** be required. Grading skills **shall** be appropriate to technology and ATA validation category used in the ocular telehealth DR program.

A licensed qualified optometrist or ophthalmologist with expertise in DR and familiarity with program technology **should** supervise readers. An adjudicating reader **may** resolve ambiguous or controversial interpretation. In most cases, an adjudicating reader **may** be an optometrist or ophthalmologist, but in all cases the adjudicating reader **shall** have special qualifications in DR by training or experience.

##### E. Information systems personnel

An information systems specialist is responsible for system privacy/confidentiality protocols, connectivity, data integrity, availability of stored images, and disaster recovery.^36,37^ The specialist **should** be available in case of system malfunction to solve problems, initiate repairs, and coordinate system-wide maintenance.

### Technical Guidelines

#### I. Equipment specifications

Telehealth systems used in the United States **shall** conform to applicable Food and Drug Administration (FDA) regulations. Telehealth systems used inside and/or outside the United States **should** meet applicable national and local statutes, regulations, and accepted standards. Elements include:

- Image acquisition hardware (computers, cameras, and other peripherals).
- Image transmission, storage and retrieval, and display systems.
- Image analysis and clinical workflow management (scheduling follow-up examinations, clinical communication management, and decision support tools).
- Security and confidentiality of protected health information (PHI) and images.

**Fig. 1.**
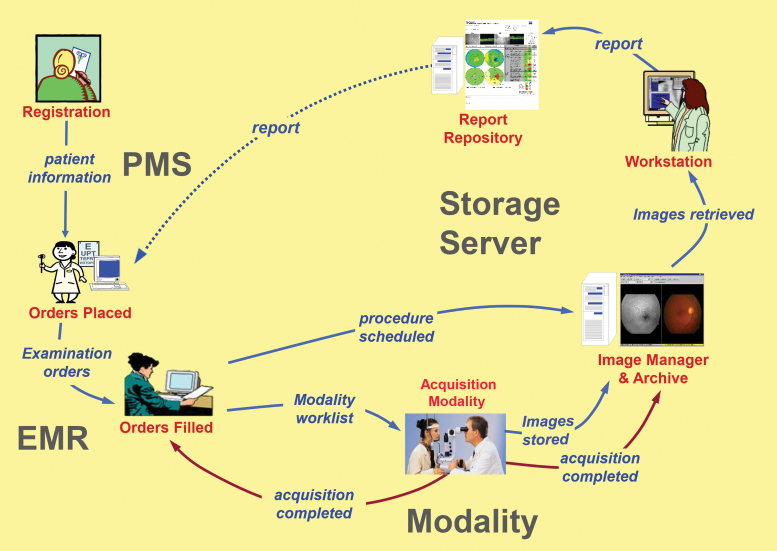
IHE-Eye Care, unified eye care workflow (Reprinted with the permission of the IHE Eye Care Domain). EMR, electronic medical record; IHE, Integrating the Healthcare Enterprise; PMS, patient management system.

Equipment specifications will vary with program needs, validation category, and available technology (*Fig. 1*). Equipment **shall** provide image quality and availability appropriate for clinical needs, program goals, and regulatory requirements. The diagnostic accuracy of any imaging system **shall** be validated as an integrated component of the overall program before incorporation into a telehealth system.^10–12,38–42^ Specific imaging and reading technology and protocols vary widely, but are generally related to the operational environment and validation category.

All relevant technologies, including image acquisition, image management/Picture Archiving and Communication System (PACS), and interfaces to patient management systems (PMSs), and electronic medical records (EMRs)/electronic health records (EHRs), **should** be Digital Imaging and Communications in Medicine (DICOM)^43^ and Health Level 7 (HL7) standards compliant. New equipment and periodic upgrades to incorporate expanded DICOM standards should be part of an ongoing performance improvement program. DICOM Supplement 91 (Ophthalmic Photography), which addresses ophthalmic digital images, was released in 2004 and updated in 2009,^44^ DICOM Supplements 110 (Ophthalmic Tomography) 2007,^45^ 173 (Wide Field Ophthalmic Photography) 2015,^46^ and 197 (Ophthalmic Tomography Angiography) 2017^47^ may be useful in certain ocular telehealth applications and **should** be considered if relevant technology is used.

##### A. Image acquisition

To provide alignment with the accepted standards for medical imaging, retinal image data sets **should** adhere to DICOM standards. When DICOM protocols are used, patient information, eye and retina characteristics, image type, type of retinal examination, retinal image set, and other data **shall** be linked to image files as metadata.^48^ Additional relevant information such as medical and surgical history, and laboratory values **may** also be included as metadata of an image series or otherwise linked to the images for use during image interpretation and reporting (*Appendix A2*).

There are many equipment options available for image capture, but most devices currently used in telemedicine for DR (Tmed-DR) are flash-based fundus cameras designed for eye clinic settings and adapted for telemedicine use.^21^ The device selected **shall** be appropriate for the program's clinical, business, and operational characteristics, and **shall** be used in a manner suitable for the validation category, and coordinated with the other equipment components of the program (see Interoperability section and *Appendix A2*).

Many factors must be considered when selecting a particular retinal imaging device and imaging protocol. Most commercially available retinal imaging devices have sufficient resolution for Tmed-DR. The minimum resolution for this purpose is 20 pixels per degree.^49^ Diagnostic accuracy of the system is the pivotal feature that enables a particular validation category. The ungradable rate is a related feature since this rate can affect the system's functional specificity.

Important features influencing diagnostic accuracy include field of view (FOV) and mydriatic versus nonmydriatic imaging.^21^ Although variation in methodology makes it difficult to compare existing reports, in general, larger aggregate FOV and mydriasis are associated with the highest diagnostic accuracy and lowest ungradable rate when using flash photography. (*Table 3*) The total FOV is the most influential feature in this consideration, with nonmydriatic ultrawide field imaging performance roughly equivalent to multifield mydriatic systems.^21^

**Table 3.** Meta-analysis of a 20-Year Review of the Telemedicine for Diabetic Retinopathy Literature

NO. OF 45° FIELDS	SENSITIVITY (%)		SPECIFICITY (%)		UNGRADABLE RATE (%)	
	MYDRIATIC	NONMYDRIATIC	MYDRIATIC	NONMYDRIATIC	MYDRIATIC	NONMYDRIATIC
1	83% ± 11	72% ± 14	88% ± 17	95% ± 4	8% ± 7	24% ± 20
2	81% ± 18	82% ± 31	92% ± 6	77% ± 23	4% ± 3	19% ± 10
3	89% ± 6	87% ± 16	93% ± 7	91% ± 14	5% ± 3	15% ± 12

The predominate format of 45° FOV systems are shown. Mean sensitivity and specificity to match program goals (diabetic retinopathy detection, severity, or referral rate) and study ungradable rate (± standard deviation) of 45° FOV systems using 1, 2, or 3 mydriatic or nonmydriatic 45° fields.

*Note:* Adapted from Horton et al.^24^

FOV, field of view.

The form factor of the imaging station (retinal camera and supporting equipment) is an early consideration during equipment selection. A system that can be easily transported between sites allows an increased and adaptable catchment area for the program while limiting equipment costs. Most retinal imaging devices for this purpose must be adapted from devices designed and marketed for conventional clinic applications.

Mobile systems based upon a smartphone platform have a favorable form factor and cost features, and carry the additional advantage of integrated image transmission. Although clinical potential has been demonstrated with these devices,^50^ limited sensitivity and specificity for DR detection and severity level diagnosis limit their use. Moreover, a lack of standardization and a short product cycle life create significant business and interoperability challenges.^51^

Portable systems using handheld imaging devices are larger and more costly than smartphones, and may suffer from some of the same limitations.^52–54^ High-quality evidence of their efficacy is lacking, although studies are ongoing to validate these devices.^55^

Another alternative for portable Tmed-DR operations is the conversion of a conventional fundus camera for portable use by use of a transportable case. This method retains the performance and connectivity benefits of the conventional retinal imager but often requires the construction of a customized hardened case for device protection, resulting in a large and heavy item that may be cumbersome to move, and requires a desktop configuration.

##### B. Image display

Retinal images used for diagnosis **should** be displayed on high-quality monitors of appropriate size, resolution, gamma setting, refresh rate, and viewing environment. Monitors, stereoscopic viewing (if applicable), and settings **should** be appropriate for the program's clinical goals, and described in its validation study. Displays **should** be calibrated regularly to ensure ongoing fidelity with original validation display conditions. Revalidation **should** be performed if settings or components are materially changed. Ambient light level, reflections, and other artifacts should be controlled in the reading area to ensure standardized viewing consistent with the original validation conditions.

##### C. Image analysis

Computer algorithms to enhance digital retinal image quality or provide automated identification of retinal pathology are emerging technologies. Image analysis tools for enhancing image quality (histogram equalization, edge sharpening, image deconvolution, etc.) or identifying lesions such as microaneurysms, hemorrhages, or hard exudates can be used to aid retinopathy assessment. Computer algorithms may also be used to facilitate and standardize reader assessment of DR and DME severity using rules based upon accepted standards. *Appendix A3* summarizes the use of autonomous and computer-assisted detection for classification and diagnosis of DR image processing.

Computer algorithms for DR assessment of retinal images **shall** undergo rigorous clinical validation with the outcome mapped to the ATA validation categories for DR before being used. Regulatory approval may be required in the United States.

The nature of telemedicine allows clinical and related patient data to be reviewed remotely in a nonclinical setting where ambient conditions and privacy are less controlled. Staff involved in assessment of Tmed-DR images and related data **shall** ensure privacy and confidentiality of all patient information. The reading environment **shall** be reasonably controlled for reader distractions, and the ambient lighting **shall** be consistent with monitor calibration.

#### II. Data management

##### A. Interoperability

Health information technology (HIT) interoperability is the ability of systems to exchange and use electronic health information from other systems without special effort on the part of the user to advance the health status of and the effective delivery of health care for individuals and communities.^56^ HIT interoperability has been recognized as a key element in moving the health care system toward improved outcomes, patient safety, and efficiencies.^57^

In the United States, an integrated digital health care system has been described by federal regulations and its implementation heavily incentivized. Initially these incentives occurred through supplemental payments, but more recently this approach has transitioned to a system of financial penalties for nonconforming providers and health care facilities. This emphasis stems from evidence that harmonized communication of HIT improves operational efficiency, patient safety, and public health reporting through the availability of patient health information at the right place and the right time. The current regulatory roadmap suggests continued regulatory attention to interoperability,^58,59^ so ocular telehealth programs **should** consider interoperability options when selecting equipment and software. Additional information about interoperability is available in *Appendix A2*.

##### B. Compression

Data compression may facilitate efficient transmission, storage, and retrieval of retinal images, and **may** be used if the algorithms have undergone clinical validation.^60,61^ DICOM recognizes lossy and lossless compression of medical images in multiple supplements relevant to ocular telehealth, and the type and character of compression used are encoded in the DICOM metadata.^44,45,62,63^ Compression types and ratios **shall** be included in clinical validation and **should** be periodically reviewed to ensure appropriate clinical image quality and diagnostic accuracy.

##### C. Data communication and transmission

A variety of technologies are available for data communication. Ocular telehealth programs **should** determine specifications for transmission technologies best suited to the program's clinical, technical, and business needs. Transmission systems **shall** have robust error checking and recovery protocols to ensure data integrity.^64^ Data communications **should** be compliant with DICOM and HL7 standards. If DICOM conformant equipment is used, ocular telehealth system equipment manufacturers **shall** supply DICOM conformance statements.

If ocular telehealth applications are integrated with existing health information systems, interoperability **should** incorporate DICOM and HL7 conformance, and establish appropriate workflow for patient scheduling and report transmission.^65^ Integrating the Healthcare Enterprise-Eye Care (IHE-Eye Care) Technical Frameworks^66^ **may** be used to further facilitate and standardize health information exchange between imaging devices and EHRs.

##### D. Archiving and retrieval

Ocular telehealth systems **shall** provide storage capacities and duration in compliance with facility, state, and federal medical record retention regulations. Images **may** be securely stored and archived locally, at imaging or reading sites, offsite, or on the web, and **shall** satisfy all jurisdiction requirements. Storage and query/retrieve transactions with PACS or other image mangers **should** conform to DICOM protocols. All study images and reports **shall** be available consistent with regulations and statues.

Each facility **shall** have digital image archiving policies and procedures equivalent to existing policies for protecting other data and hardcopy records. Telehealth programs **shall** also address HIPAA security requirements for data backup and archive.

##### E. Security

Ocular telehealth systems **shall** have network and software security protocols to protect patient confidentiality and identification of image data. Measures **shall** be taken to safeguard and ensure data integrity against intentional or unintentional data corruption. Privacy **should** be ensured through a minimum 128-bit encryption and two-factor authentication technology. Digital signatures **may** be used at image acquisition sites. Transmission of retinal imaging studies and study results **shall** conform to HIPAA privacy and security requirements.

##### F. Reliability and redundancy

Written policies and procedures **shall** be in place to ensure continuity of care and conformance to HIPAA requirements at levels similar to that for hardcopy retinal imaging studies and medical records. Policies and procedures **should** include internal redundancy systems, backup telecommunications, and a disaster recovery plan. Ocular telehealth reports **shall** be retained and digital retinal images **should** be retained as part of patient medical records in a manner and duration to meet regulatory, facility, and medical staff clinical needs.

##### G. Documentation

Readers rendering reports on DR or other ocular abnormalities **should** comply with standardized diagnostic and management guidelines as established by the American Academy of Ophthalmology^67^ or the American Optometric Association.^68^ Reports **should** be based on HL7 or DICOM formats to facilitate health information exchange and recognition by quality performance surveys. Reports **should** provide DR severity levels consistent with accepted standards as appropriate for ATA validation category used. Medical nomenclature **should** conform to Systematic Nomenclature of Medicine Clinical Terms (SNOMED CT)^69^ standards. Transmission of reports **shall** conform to HIPAA privacy and security requirements.

### Administrative Guidelines

#### I. Legal requirements

Legal and regulatory issues relating to the practice of ocular telehealth are generally the same as other telemedicine modalities and carry the risk management considerations of conventional medical practice.^33,70,71^ A DR telehealth program **should** use the same safeguards to mitigate risk.

##### A. Facility accreditation

Some hospital telehealth programs fall within regulatory jurisdictions of The Joint Commission (TJC) and/or Centers for Medicare and Medicaid Services (CMS).^72^ TJC and Accreditation Association for Ambulatory Health Care accredit ambulatory health care facilities.^73,74^ These accrediting bodies publish standards that apply to telemedicine activities, making regulatory compliance a mandatory component for most hospital-based telehealth programs. There are specific references to telemedicine in TJC Environment of Care and Medical Staff sections, including LD.04.03.09, MS.13.01.01, and MS.13.01.03.^75^

CMS requirements also occur indirectly through related activities, such as standards for contract care. There are other accreditation standards that may apply to a specific program and clinical setting, with similar, but not identical requirements. Awareness and understanding of these standards and the applicable CMS regulations can be daunting.^76^ Ocular telehealth programs **shall** carefully review applicable standards to ensure conformance.

##### B. Health Insurance Portability and Accountability Act

Ocular telehealth programs **should** obtain professional consultation for HIPAA compliance specific to their program. Telehealth programs **shall** consider HIPAA privacy^77,78^ and security^79,80^ regulations in clinical, administrative, and technical operation plans. Privacy and security issues are listed in *Appendix A4*.

##### C. Privileging and credentialing

Ocular telehealth providers **may** require privileging and credentialing. Licensed providers responsible for interpretation of retinal telehealth images **shall** be credentialed and obtain privileges at OS and DS if required by applicable statues and regulations, and facility bylaws.^81,82^ Technical staff usually do not require formal privileging and credentialing, but **shall** have their duties and job-specific competencies described in a position description or equivalent. If telemedicine providers undergo credentialing and privileging, ocular telehealth programs **should** utilize the CMS regulations and accreditation standards for “privileging and credentialing by proxy.” See *Appendix A5* for CMS regulations and accreditation standards for telemedicine providers.

##### D. Fraud and abuse

Telemedicine programs are subject to the fraud and abuse statutes and regulations concerning health care-related kickbacks and other financial inducements for referrals. The antikickback statute prohibits payment or any receipt of remunerations for referrals or purchasing equipment reimbursable under federal health programs.^83^ The language in this law is so broad that “Safe Harbors” were created to lessen the impact on legitimate ventures.^84^

The Stark Act prohibits physicians from making a referral for designated health services to an entity with which the physician (or immediate family member) has a financial relationship.^85,86^ Self-referrals occur when physicians refer patients to medical facilities in which they or their immediate family have a financial interest. For example, an ophthalmologist places a retinal imaging workstation in a primary care provider's office at deep discount or gratis and reads images at little or no charge. The Stark statute may have been violated if patients needing treatment are referred to the ophthalmologist. This practice may be avoided by charging the primary care provider full market value for equipment and services and offering the patient a choice of referral ophthalmologists for treatment.^87^

Ocular telehealth programs **should** obtain council to establish policies and operational practices that prevent violation of the antikickback laws and Stark Act.

Another area of risk under the general category of fraud and abuse is antitrust. Although telemedicine and other e-health practices offer the opportunities of improved business efficiencies, reduced incremental costs of services, and new product offerings, in certain settings they may also be interpreted as restraining trade. To mitigate antitrust risks, the ocular telehealth program **should** identify aspects of the program that threaten competition and implement appropriate safeguards under the guidance of council.

##### E. State medical practice acts/licensure

In general, telehealth legal issues assume telemedicine is the practice of medicine, and telemedicine and telehealth programs are subject to the ordinary laws and regulatory oversight that govern all medical providers. These issues are addressed variably by state medical practice acts, but even in the absence of specific statutory or regulatory definitions, telehealth legal claims would be difficult to defend against otherwise.^33^

All 50 states, the District of Columbia, and the U.S. territories require licensure for rendering medical care to patients located in their jurisdiction, and a physician is considered subject also to the medical practice laws and regulations where the patient is located. Many states provide for some degree of telemedicine-friendly licensure or license “portability” for telemedicine, including a small number of states with telemedicine or special purpose licensure, and a larger number with participation in the Interstate Medical Licensure Compact.^88^ This compact allows qualified physicians seeking to practice in multiple states to be eligible for expedited licensure in all states participating in the compact.

The ATA Interstate Telehealth Special Interest Group (SIG) is a source of current information on cross-border practice developments.^89^ Since this is an active topic for legislative attention in many states, all programs **should** closely examine the licensure options in states of intended practice.^90,91^

##### F. Tort liability

Telemedicine may reduce overall liability risks through improved access and quality of care and improved documentation. However, experience indicates that telemedicine may increase the risk for liability for providers and facilities that use it and for those who chose to not use it. The elements of a medical malpractice claim are well established, but telemedicine can also complicate traditional tort liability. Issues include which entity or physician owes a duty to the patient, standards-of-care, jurisdiction, and choice of law.^33^ Although telemedicine providers should consult an attorney familiar with telemedicine law, the fundamental aspects of tort law are fairly uniform across jurisdictions:

- A physician has a duty to a patient to act within the accepted standards-of-care.
- Standards-of-care were violated.
- A patient suffered an injury due to the violation of standard-of-care.

1. Duty

A physician's duty arises from the physician–patient relationship.^92^ Telemedicine alters the traditional context of this relationship but a telemedicine encounter is sufficient to establish the relationship.^33,93^

2. Standards-of-care

The American Medical Association believes medical specialty societies should develop or participate in the development and implementation of telemedicine clinical guidelines and position statements.^94^ Because telemedicine standards-of-care are not universally established and recognized, questions could arise regarding appropriateness of a telemedicine DR evaluation, whether appropriate technology was selected (e.g., Validation Category 1, 2, 3 or 4), or whether the outcome was appropriate for a particular setting or case. An example of a controversial outcome is failure to diagnose nondiabetic retinopathy pathology evident in images (e.g., venous occlusion and choroidal neovascular membrane [CNVM]), or not evident in images (e.g., choroidal melanoma anterior to the equator and peripheral retinal tear/retinal detachment).

Issues of jurisdiction, choice of laws, and apportionment of liability are additional issues that are incompletely defined by statute and case law.^95^ Telehealth providers **should** consult with legal counsel and their professional liability carrier to ensure proper risk management and medical liability coverage in both OS and DS.

##### G. Consent

Patients have the right to autonomous informed participation in health care decisions,^96^ but this right cannot be exercised without enough information to allow an informed choice.^97^ Informed consent is required for clinical treatments and procedures, including those delivered through telemedicine. When treatments or procedures delivered through ocular telehealth are considered low risk and within commonly accepted standards of practice, oral consent may be sufficient and a written and signed consent may not be required.^81^ Ocular telehealth services for DR may satisfy these criteria. Patients **should** be informed that they have a choice of telehealth and nontelehealth ocular assessment, treatments, or procedures. Practitioners **should** provide patients' information about the ocular telehealth program they would reasonably want to know, including:

- Whether the services is novel or experimental.
- Differences between care delivered using ocular telehealth and face-to-face examination.
- Benefits and risks of using ocular telehealth in the patient's situation.
- Description of what is to be done at the patient's site and the remote site.

Informed consent requirements vary from state to state, and currently, only a few states have laws that mandate informed consent for telemedicine treatment. However, ocular telehealth providers and programs **should** consult the statutes in their jurisdiction to determine whether oral or written informed consent is required for the telehealth services they render.

#### II. Quality control

A structured process for quality control and ongoing performance improvement is fundamental in health care,^98^ and no less so for Tmed-DR. A codified method for collection, analysis, and reporting of programmatically relevant data **must** be used to document clinical and programmatic outcomes, and ensure patient safety, regulatory compliance, patient and provider satisfaction, and program sustainability. This process is necessarily end-to-end in scope, defined by specific and quantifiable quality measures relevant to the program, and must include a process for correction of identified fallout.

The technical quality of images and completeness of associated clinical data **shall** be assessed on an ongoing basis to ensure that their interpretation meets specified standards. Policies **must** be in place to ensure patient care and safety,^70,99,100^ including addressing non-DR eye diseases and findings not specifically related to DM. Ocular telehealth programs **shall** also develop protocols that include policies and procedures for monitoring and evaluating performance.^81^

Corrective action of undesired trends and context-sensitive continuing education (CE) **shall** be included. Evaluation **shall** be tailored to include all components, such as image acquisition, transmission, reading and reporting, as well as related features such as reading latency, reporting duration, and referral completion. Image acquisition and reading quality assessment and performance improvement are similar to clinical settings. Quality assessment **shall** measure staff performance, data quality, and workflow. In the case of licensed providers, peer review of clinical outcome and identification of fallout cases to guide corrective interventions **shall** be performed in alignment with local policy and accreditation requirements.^101,102^ Training and education standards **shall** be developed. An example of performance categories and measures, and training and QA methods is included in *Appendix A6*.

#### III. Operations

An operations manual is a comprehensive documentation of how a program functions on a daily and ongoing basis. A DR operations manual contains operational information and description of key processes in sufficient detail to provide standardized performance at all levels of the program, and also guide new leadership and staff. It can also describe QA and staff training procedures, but is not intended to function as an employee handbook. A comprehensive manual enables normal operations during leadership absence, and provides a pathway to programmatic sustainability during staffing changes at any level. Ocular telehealth programs **should** develop and implement an operations manual that is dynamic and evolves to remain aligned with program methods and goals.

#### IV. Customer support

Ocular telehealth programs use advanced technology in a broad range of settings, operated by diverse staff with varying training and expertise. Ocular telehealth programs **should** have a structured support system tailored to meet the needs of both internal and external customers. This support can be categorized by:

##### A. Originating site

- Imager: imaging process, hardware/software, initial training and provisional certification, recurrent training, QA, and evidenced-based recertification.
- Imaging device: image acquisition, operator-based service, device faults, preventive maintenance, site-based calibration, and diagnostics.
- Provider/clinical contact: report retrieval/delivery and interpretation, patient recall, and billing.

##### B. Data transmission

- Connectivity/network errors.
- Data loss/recovery.

##### C. Distant site

- Reader adjudication, initial training and provisional certification, QA/peer review, recurrent training, and evidence-based recertification.
- Diagnostic display equipment and software.

OS and DS may be in the same facility with data transmission contained within a single local area network. Support for such systems is typically less complex than geographically distributed programs involving multiple networks and servers. Technical support can be divided into levels, or tiers, depending on difficulty or urgency. Tiered help desks are common and a convenient way to accommodate program needs efficiently. A DR telehealth program **should** establish standards for addressing customer support needs and tracking resolution of operational and technical problems. The outcome of customer support **should** be a routine component of the program's larger QA program. *Appendix A7* provides examples of support levels and support priority.

#### V. Financial factors

Telehealth program sustainability depends on a well-developed and executed business plan. The actual cost of services can be a complex calculation, and reimbursement depends upon accurate diagnostic and procedural coding, and pays for performance and quality incentives. The specifics of these issues vary between regions, payers, and clinical settings, so each program should tailor billing protocols with Medicare, Medicaid, and private insurance intermediaries.

##### A. Reimbursement

Billing codes and reimbursement coverage are pivotal components for successful reimbursement. Billing is usually divided into technical or image capture (Current Procedural Terminology [CPT] suffix TC) and professional or interpretation components (CPT suffix 26). Before 2011, most DR telehealth programs used the 92250 (Fundus Photography with Interpretation and Report) CPT code. Infrequently, programs used CPT 92499 (Unlisted Ophthalmic Service or Procedure), which requires negotiated use with the fiscal intermediary or carrier.

In 2011, the CMS approved two new codes specific for remote retinal imaging, CPT 92227 and 92228. The reimbursement landscape is highly dynamic, and has substantial state, regional, and payer differences. Failure to attend to these changing differences appropriately can result in failed reimbursement and, in some instances, costly penalties. For these reasons, programs **should** seek ongoing council to ensure compliance with the requirements of a particular payer or fiscal intermediary, and locale. See *Appendix A8* for additional information regarding billing and reimbursement of DR ocular telehealth services.

##### B. Grants

Grants have been used to establish telemedicine programs for defined circumstances and duration. Although an important method for proof of concept, grants are usually not viable for sustained clinical operation. As telehealth programs become more common as routine tools for health care, grants have become less common business plans for DR ocular telehealth. DR telehealth programs **should** have business plans that ensure revenue for sustainability, usually through reimbursement for services through Medicare, Medicaid, private insurance carriers, or per capita or transaction-based contracts.

##### C. Federal programs

There are several large telemedicine programs that reside within federal agencies and are funded by recurring federal appropriations. Examples include the Indian Health Service and the Veterans Health Administration. These programs sometime supplement their federal appropriations with external reimbursements, but their predominant business plan is cost avoidance through improved outcomes stemming from increased compliance with standards-of-care.

##### D. Other financial factors

Nonrevenue financial benefits of a DR telehealth program may include cost savings over traditional care delivery; however, benefits may not be realized by the entity creating them. For example, patients and third-party payers may realize financial savings through cost avoidance produced by a DR telehealth program, whereas the primary care physicians funding the program realize little or no direct savings.

Under current reimbursement policies in the United States, DR telehealth may be a better business model in closed systems, such as managed care, where costs and return on investment are realized by the same entity that funds and operates the program. However, it is important to recognize that cost-avoidance benefits occur over time and may not immediately offset day-to-day operational expenses. Government pay-for-performance incentive programs may change the relationship between program funding and reimbursement in the future. *Appendix A8* contains financial information on logistic efficiencies, disease prevention, and resource utilization.

##### E. Equipment cost

Imaging costs depend on many factors, but with the decreasing cost of computing and telecommunications, a retinal camera is frequently the largest capital investment for a DR telehealth program. Retinal imaging devices range from $3,500 to >$85,000, including fundus camera, camera back, auxiliary lenses, computer, software, and network hardware. Almost all retinal imagers used by ocular telehealth DR programs are adaptations of devices designed for conventional eye clinic use. Consequently, they have technical and operation features and price points that are not optimized for the telehealth setting. The specific imaging device selected for a particular ocular telehealth DR program depends on its target clinical goals, business plan, clinical design, and other factors tied to clinical outcome and program scalability and sustainability. An ocular telehealth DR program **should** carefully weigh these factors before selecting a specific retinal imager.

Abbreviations

AEs: adverse events
AI: artificial intelligence
AMD: age-related macular degeneration
AREDS: Age-Related Eye Disease Study
ATA: American Telemedicine Association
BIO: binocular indirect ophthalmoscopy
CAH: critical access hospital
CBIA: computer-based image analysis
CE: continuing education
CMS: Centers for Medicare and Medicaid Services
CNNs: convolutional neural networks
CNV: choroidal neovascularization
CNVM: choroidal neovascular membrane
CoP: conditions of participation
CPT: Current Procedural Terminology
CQI: continuous quality improvement
CSME: clinically significant macular edema
DICOM: Digital Imaging and Communications in Medicine
DM: diabetes mellitus
DME: diabetic macular edema
DR: diabetic retinopathy
DS: distant site
EHRs: electronic health records
EMR: electronic medical record
ETDRS: Early Treatment Diabetic Retinopathy Study
FDA: Food and Drug Administration
FOV: field of view
GA: geographic atrophy
HE: hard exudates
HIPAA: Health Insurance Portability and Accountability Act
HIT: health information technology
HL7: Health Level 7
IHE: Integrating the Healthcare Enterprise
LIP: licensed independent provider
MACs: Medicare Administrative Contractors
NICU: neonatal intensive care unit
NPDR: nonproliferative diabetic retinopathy
OCT: optical coherence tomography
OS: originating site
PACS: Picture Archiving and Communication System
PDR: proliferative diabetic retinopathy
PHI: protected health information
PMS: patient management system
QA: quality assurance
QALY: quality-adjusted life year
RFP: request for proposal
RNFL: retinal nerve fiber layer
ROC: receiver operating characteristics
ROP: retinopathy of prematurity
RVUs: relative value units
RW-ROP: referral-warranted ROP
SIG: Special Interest Group
SNOMED CT: Systematic Nomenclature of Medicine Clinical Terms
SNOMED: Systematized Nomenclature of Medicine
STDR: sight-threatening diabetic retinopathy
SUNDROP: Stanford University Network for Diagnosis of Retinopathy of Prematurity
TJC: The Joint Commission (formerly Joint Commission for the Accreditation of Healthcare Organizations)
Tmed-DR: telemedicine for DR
TRCs: Telehealth Resource Centers
VEGF: vascular endothelial growth factor

## Glossary

DICOM: Digital Imaging and Communication in Medicine.

An international standard for distributing, storing, and viewing medical images.

HL7: Health Level 7.

An international framework for the electronic exchange of clinical, financial, and administrative information among computer systems in hospitals, clinical laboratories, pharmacies, etc.

IHE: Integrating the Healthcare Enterprise.

A global initiative by health care professionals and industry to improve computer sharing of health care information through coordinated use of established standards such as DICOM and HL7.

SNOMED CT^®^: Systematic Nomenclature of Medicine Clinical Terms.

A system of clinical health care terminology covering diseases, findings, procedures, microorganisms, pharmaceuticals, etc.

## Figures and Tables

**Fig. 2.**
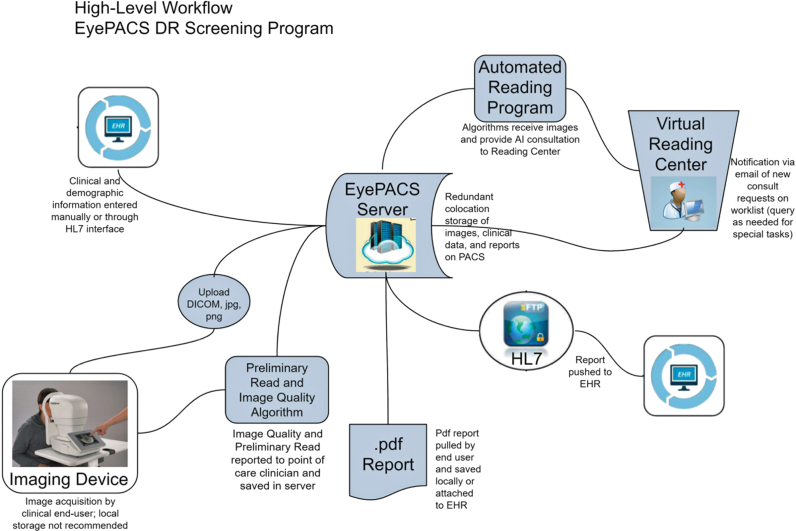
Example of web-based image management, reading center, and report delivery (Public domain. Courtesy EyePacs, San Jose, CA). EHR, electronic health record; HL7, Health Level 7; PACS, Picture Archiving and Communication System.

**Fig. 3.**
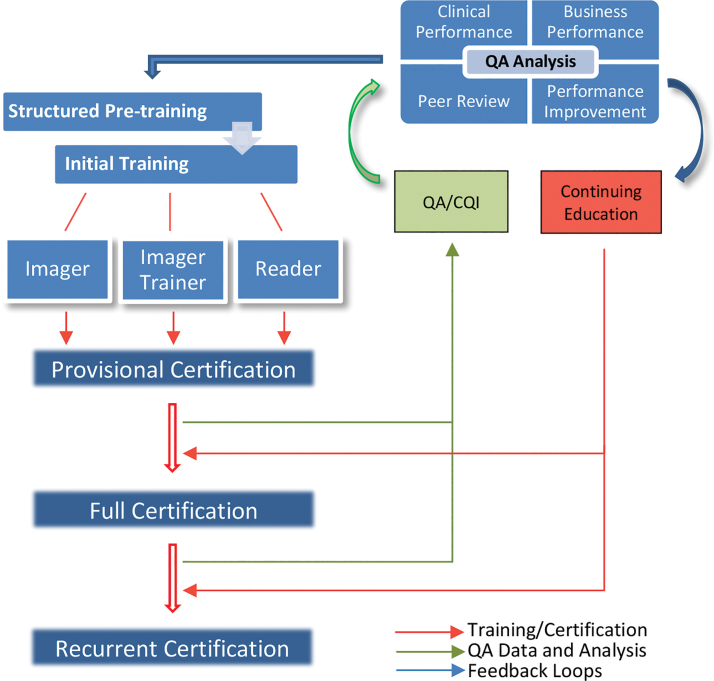
QA and CQI flow diagram. CQI, continuous quality improvement; QA, quality assurance.

## Appendices

### Appendix A1. Clinical Validation

As telemedicine programs for DR were first developing, there was considerable debate about technical requirements, such as the number and field size of images needed for evaluation, the need for color versus gray scale images, stereoscopic viewing, and image compression. Early telehealth programs for DR varied considerably with respect to their technology, operational features, and clinical outcomes. In an effort to facilitate provider and patient expectations, standardized reporting, and program development, the ATA published *Telehealth Practice Recommendations for Diabetic Retinopathy* in 2004^103^ through a collaboration between the ATA Ocular Telehealth SIG and the National Institute of Standards and Technology.

The ATA practice recommendations for DR were predicated, in part, upon earlier work to standardized classification and treatment of DR. In 1967, a group of leading clinicians and researchers met at the Airlie House in Virginia to address the growing problems of blindness and vision loss from DR. One outcome of the meeting was the Airlie House Classification of Diabetic Retinopathy. This classification was subsequently modified by the Diabetic Retinopathy Study^104^ and expanded by the ETDRS.^6^

The classification relied on a group of standard photographs that illustrated characteristic lesions to grade a level of DR. To grade the level of DR, the presence and degree of these lesions were assessed in seven 30° retinal fields, and the classification, with minor modifications and expansion, became the grading tool for major clinical trials worldwide. Reference to these standard photographs supported the ETDRS classification of DR.^6^

Since the Diabetic Retinopathy Study and the ETDRS provided firm evidence-based treatments for DR, it was crucial that ocular telehealth programs for DR perform within the standards established by these and other studies. The Airlie House Classification of Diabetic Retinopathy was chosen for program validation by the ATA.

### Telehealth Practice Recommendations for Diabetic Retinopathy

Telehealth programs for DR should clearly define program goals and program performance in relation to accepted clinical standards. In general, the selection of an ocular telehealth system for evaluating DR should be based on the unique needs of the program's health care setting.

The *Telehealth Practice Recommendations for Diabetic Retinopathy* recognized four categories of validation for telehealth for DR using ETDRS 30°, stereo seven-standard fields, color, 35 mm slides (ETDRS photographs) as a reference standard. Since grading scales and standards other than ETDRS are in use for grading DR,^14^ DR telehealth programs should state the standards used for validation and relevant data sets used for comparison. Furthermore, although ETDRS photographs are a well-established standard for evaluating DR, there is no clear-cut consensus on a suitable digital photography protocol as a replacement for ETDRS photographs, although ongoing clinical trials are investigating various imaging devices and techniques. With the advent of digital photography and the migration away from film photography, digital retinal images have become the norm for major clinical trials.^7^

Until standards for digital imagery are firmly established, telehealth programs for DR should demonstrate an ability to compare favorably with ETDRS photographs or a suitable alternative as reflected in kappa values for agreement of diagnosis, false-positive and false-negative readings, positive predictive value, negative predictive value, sensitivity and specificity of identifying referral thresholds of DR severity, and macular edema. The inability to obtain or read images (ungradable images) should be considered a positive finding for disease in telehealth programs for DR, and persons with unobtainable or ungradable images generally should be referred for evaluation by an eye care specialist.

The validation categories are not determined by any component of image capture or image review, but rather are an outcome of the overall program in an end-to-end manner. These categories were defined in the original *Telehealth Practice Recommendations for Diabetic Retinopathy*^103^ and refined in the third edition as follows:

#### Category 1

Category 1 validation indicates a system can separate patients into two categories: (1) those who have no or very mild NPDR (ETDRS level 20 or below) and (2) those with levels of DR more severe than ETDRS level 20. Functionally, category 1 validation allows identification of patients who have no or minimal DR and those who have more than minimal DR.

- Clinical Performance: the system can distinguish patients with no or very mild NPDR from those with levels greater than very mild NPDR.
- Clinical application: screen for presence versus absence of DR.

#### Category 2

Category 2 validation indicates a system can accurately determine whether STDR as evidenced by any level of DME, severe or worse levels of NPDR (ETDRS level 53 or worse), or PDR (ETDRS level 61 or worse) is present or not present. Category 2 validation allows identification of patients who do not have STDR and those who have potentially STDR. These patients with STDR generally require prompt referral for possible laser surgery.

- Clinical performance: the system can distinguish patients with moderate or less NPDR and no DME from those with NPDR greater than moderate or any level of PDR, or any level of DME.
- Clinical application: screen for presence versus absence of potentially STDR.

#### Category 3

Category 3 validation indicates a system can identify ETDRS-defined levels of NPDR (mild, moderate, or severe), PDR (early and high risk), and DME with accuracy sufficient to determine appropriate follow-up and treatment strategies. Category 3 validation allows patient management to match clinical recommendations based on clinical retinal examination through dilated pupils.

- Performance: the system can distinguish patients with ETDRS-defined clinical levels of NPDR (mild, moderate, or severe), PDR (early and high risk), and DME (central-involved DME or not central-involved DME).
- Clinical application: match clinical recommendations based on conventional clinical retinal examination through dilated pupils or ETDRS photographs, allowing remote management of DR.

#### Category 4

Category 4 validation indicates a system matches or exceeds the ability of ETDRS photographs to identify lesions of DR to determine levels of DR and DME. Functionally, category 4 validation indicates a program can replace or coexist with ETDRS photographs in any clinical or research program.

- Performance: the system can identify the presence and degree of specific DR lesions to match the ability of ETDRS photographs to determine all specific lesions and ETDRS levels of DR and DME.
- Clinical application: replace or coexist with ETDRS photographs as a gold standard in any clinical or research program.

The current guidelines continue with the original basic validation categories for telemedicine programs for DR. Since the current evidence shows great heterogeneity in the methods of program testing and reporting, the following guidelines were added to improve the scientific rigor of validation studies and standardization of program description.

- Validation of a category should assess a program's “end-to-end” performance and not any single piece of its technology, imaging protocol, or grading protocol.
- The study design to validate a program should follow conventional scientific methodology and apply appropriate statistical rigor. Many programs have published the results of validation studies in the peer-reviewed literature, but this has become less practical with the proliferation of telehealth programs for DR. In the future, a clearing house for validation studies may exist to provide an external review of validation study method and outcome. Until then, programs should independently seek an unconflicted external review of their validation study.
- The validation study cohort must include appropriate representation of all severity levels of DR from none through PDR, and none through clinically significant/central-involved DME.
- The method and accuracy of detecting and risk-stratifying DME using other than direct measures, for example, stereoscopic viewing and OCT, should be carefully described. Referral thresholds for DME should be appropriately reflected by the validation study outcome.
- Although ETDRS photographs currently provide an ideal standard for validation, clinical comparators may be used for program validation if the examination is conducted by a retinal specialist using accepted best practices.

These categories should not be considered a quality continuum, but rather performance categories that describe distinct clinical outcomes of public health relevance, reflecting program goals and operating capability. In addition, they provide a standardized language for communicating performance for clinical, research, reimbursement, and regulatory compliance purposes. Information about the program's validation study design and performance should be publicly available to users and other stakeholders. Programs contracting for DR telemedicine services should consider inclusion of ATA validation category or other validation outcome language in RFPs and contract scope of work.

### Appendix A2. Interoperability

In a fully developed form, standards-based interoperability provides the free exchange of health data and associated demographics among information systems and devices in a vendor neutral manner. Increasingly, the benefits of an integrated and interoperable EHR have become an expectation of patients, providers, payers, and regulators.

Since the integration of HIT occurs on several levels, more than one definition of “interoperability” must be considered even though *imaging device to EHR* and *imaging device to image manager* are the predominant use cases in the ocular telehealth domain. Implementation of technical frameworks for interoperability is variable among software and imaging hardware manufactures, and many installed legacy devices lack the software platforms needed for standards-based exchange of health care data. These challenges must be overcome to satisfy the long-term plans described by federal regulators.^58^

Standardized terminology, software, and communication protocols are required to allow efficient interconnections to occur between devices, EHR, and practice management systems in a nonproprietary manner.^59^ Similarly, harmonization of these standards is needed to allow efficient information exchange between systems with interoperable use of data by devices and software from different vendors (*Figs. 1 and 2*).^105^

Program-level interoperability provides data that may be subsequently shared between other systems and networks to accomplish broad exchange of patient health information by public and private entities using standards-based protocols.^106^

DR ocular telehealth systems **should** include nonproprietary interoperability by using components that conform to:

- DICOM.^43^
- HL7.^72^
- IHE-Eye Care.^66^
- SNOMED CT.^69^
- Logical Observation Identifiers Names and Codes.^107^

DICOM files contain the images obtained by ocular telehealth devices and image metadata important for interpretation of exchanged files. The following are examples of key data and their parenthetical codes in the DICOM metadata.

Demographics

- Patient name (0010, 0010).
- Medical ID number (0010, 0020).
- Patient birth date (0010, 0030).
- Gender (0010, 0040).
- Date and time of examination (0008, 0020) and (0008, 0030).
- Name of facility or institution of acquisition (0008, 0080).
- Accession number (0008, 0050).
- Modality or source equipment that produced the ophthalmic photography series (0008, 0060).
- Referring physician's name (0009, 0090).
- Manufacturer (0008, 0070).
- Manufacturer model name (0008, 1090).
- Software version (0018, 1020).
- Station name (0008, 1010).

Examples of examination information in the DICOM standard for ophthalmic photography (Suppl. 91 OP):

- Image type or image identification characteristics (0008, 0008).
- Instance number or image identification number (0020, 0013).
- Mydriatic (pupil dilation) or nonmydriatic (no pupil dilation) imaging. Pupil dilated Yes/No (0022, 000D), dilating agent (0022, 001C).
- Size of field or horizontal FOV in degrees (i.e., 20°, 30°, 45°, 50°, 60°, and 200°) (0022, 000B).
- Identification of single retinal field images, simultaneous or nonsimultaneous stereo pairs.
- Identification of stereo pairs. Left image sequence (0022, 0021), right image sequence (0122, 0022).
- Monochrome gray scale or color bit depth, ophthalmic photography 8-bit images (0028, 0100, 0028, 0101), 16-bit images (0028, 0102).
- Laterality of eye, right, left, or both eyes; OD, OS, or OU (0020, 0062).
- Retinal region such as ETDRS fields 1 to 7 (0008, 0104).
- Ratio and type (i.e., wavelet or Joint Photographic Experts Group) of compression, if used. Lossy compression Yes/No (0028, 2112), lossy compression ratio (0028, 2112), and lossy compression method (0028, 2114).
- Detector type, CCD or CMOS (0018, 7004).
- Spatial resolution of the image (i.e., 640 × 480, 1000 × 1000, etc.).
- Free text field for retinal imager study comments (presence of media opacities, poor fixation, poor compliance, etc.).
- Description of any image postprocessing.
- Measurement data and/or pixel spacing (0028, 0030).

Conformance to open standards enables, but does not ensure, interoperability. Also, interoperability of EMRs, EHR, and personal health records may not always be possible or practical. However, when technical frameworks for standards-based interoperability exist, teleophthalmology programs **should** utilize them to improve operational efficiency, data integrity, patient safety, and regulatory compliance.

### Appendix A3. Automated and Computer-Assisted Detection, Classification, and Diagnosis of Diabetic Retinopathy

Michael D. Abramoff, MD, PhD,^1–6^ Theodore Leng, MD, MS,^7,8^ Daniel SW Ting, MD, PhD,^9,10^ Kyu Rhee, MD, PhD,^11^ Mark B. Horton, OD, MD,^12^ Christopher J. Brady, MD, MHS,^13^ and Michael F. Chiang, MD^14,15^

^ 1^Department of Ophthalmology and Visual Sciences, The University of Iowa, Iowa City, Iowa.

^ 2^IDx, Coralville, Iowa.

^ 3^Stephen A. Wynn Institute for Vision Research, The University of Iowa, Iowa City, Iowa.

 Departments of ^4^Biomedical Engineering, and ^5^Electrical and Computer Engineering, The University of Iowa, Iowa City, Iowa.

^ 6^Iowa City VA Health Care System, Iowa City, Iowa.

^ 7^Byers Eye Institute, Stanford University School of Medicine, Palo Alto, California.

^ 8^Spect, Inc., San Francisco, California.

^ 9^Singapore National Eye Center, Singapore Eye Research Institute, Singapore, Singapore.

^10^Duke-NUS Medical School, National University of Singapore, Singapore, Singapore.

^11^IBM Watson Health, Cambridge, Massachusetts.

^12^Phoenix Indian Medical Center, Phoenix, Arizona.

^13^Larner College of Medicine, University of Vermont Medical Center, Burlington, Vermont.

^14^Department of Ophthalmology, Casey Eye Institute, Oregon Health and Science University, Portland, Oregon.

^15^Department of Medical Informatics and Clinical Epidemiology, Oregon Health and Science University, Portland, Oregon.

### Introduction

Systems for computer-assisted and fully automated detection, triage, and diagnosis of DR from retinal images show great variation in design, level of autonomy, and intended use. Moreover, the degree to which these systems have been evaluated and validated is heterogeneous. We use the term DR AI system as a general term for any system that interprets retinal images with at least some degree of autonomy from a human grader.

### Rationale

The introduction of AI in medicine has raised significant ethical, economic, and scientific controversies. Because an explicit goal of AI is to perform processes previously reserved for human clinicians and other health care personnel, there is justified concern about the impact on patient safety, efficacy, equity and liability, and the labor market.

To partially address these controversies, the partnership on AI was established to formulate best practices for the application of AI technologies, to advance the public's understanding of AI, and to serve as an open platform for discussion and engagement about AI and its influences on individuals and society.^108^ More recently, the American Medical Association, in a series of policy statements, most recently in 2019, has been addressing these concerns for the health care field, such as the formulation of the principles of safety, efficacy, and equity of AI, and autonomous AI, in both design and validation, the integration of AI into the health care system, and when the AI developer should assume liability, as well as the development of a common nomenclature and guidelines for domain-specific systems.^109^

If AI systems in general, and DR AI systems specifically, are to gain acceptance by patients, medical providers, payers, and the general public, a common language for describing them widely agreed upon guidelines, and the upholding and dissemination of these principles are essential. This appendix is intended to establish a common framework and lexicon for consideration of DR AI systems, and provide a starting point for future practice guidelines. In this context, the following discussion will refer to these preliminary recommendations as “guidelines.”

Currently, most AI systems function as augmented intelligence, wherein there is a combination of human tasks that are difficult or impossible to computerize (e.g., common sense, morals, compassion, imagination, and abstraction) and AI system tasks (e.g., pattern identification and machine learning) to achieve high clinical accuracy, low intraobserver variability, and improved system scalability.^110^ In the case of DR AI systems, a fully automated grading of a retinal photograph to identify a threshold-level DR may allow a provider or program to determine whether referral to an eye care provider is needed as a component of the patient's diabetes care.^111^ With sufficient clinical accuracy, cost, and ease of use, multiple other use cases both inside (e.g., optometric or ophthalmogic office, and fundus reading center) and outside (e.g., pharmacy and phlebotomy laboratory) traditional eye care may also find value in such systems.

In these guidelines, the following components of an AI system will be discussed in sequence: “Level of Device Autonomy,” “Intended Use,” “Level of Evidence for Diagnostic Accuracy,” and “System Design.” At the current stage of scientific and legal evidence, there is no basis for recommending a specific combination of autonomy, accuracy, and intended use as more appropriate than any other. Thus, the current guidelines treat each component as independent, with the practice recommendations necessarily descriptive, rather than prescriptive. Issues such as patient recruitment, patient referral, and the wider health care context in which these operate are outside the scope of these guidelines.

Where possible, these practice recommendations align with other published guidelines, including the FDA's proposed *Software as A Medical Device (SaMD): Clinical Evaluation Guidelines*,^112^ and presentations by FDA after their recent authorization of the first autonomous diagnostic AI.^111^ It is to be expected, as our understanding of DR AI systems advances, that these guidelines may become more prescriptive. Since DR AI systems are a relatively new introduction in health care, many readers may not be familiar with their associated lexicon, categorical structure, and quality measures. These and other features of this type of software that operates as a medical device have been described by the International Medical Device Regulators Forum, and may facilitate understanding of these guidelines.^112^

### Level of System Autonomy

The autonomy of DR AI systems is categorized in reference to the diagnostic decision being made by the DR AI. In other words, the autonomy levels reflect the level of (or lack of) expert oversight of the clinical decision in clinical care. Autonomy levels in reference to the patient decision are (1) no autonomy, (2) assistive, or (3) autonomous, and are provided in *Table 4*.

**Table 4.** Description of Artificial Intelligence System Levels of Autonomy

AUTONOMY LEVEL	NO AUTONOMY	ASSISTIVE	AUTONOMOUS
Description	System that does not provide treatment, diagnosis, or screening recommendations	AI system that assists clinicians by giving treatment, diagnosis, or screening recommendations, while relying on physician interpretation of said advice to direct patient care	AI system that provides direct treatment or diagnosis/screening recommendations without physician interpretation

Classifying DR AI systems according to autonomy level has implications for patient safety, testing/validation, and, therefore, the claims that can be made about such a system. The more autonomously an AI system operates, the higher the requirements are for technical sophistication, validation, and system controls. The FDA has authorized or cleared systems in each autonomy level.

#### Intended Use

The purpose of this section is to put forth a classification system for specifying the intended use of any system for the detection, triage, or diagnosis of DR from images, including DR AI systems. In this discussion, *intended use* refers to the planned sociotechnical environment of the users and patients.^113^ There are multiple characteristics of intended use, the most prominent being the operational environment, type of output, and end user. Although this intended use classification system is proposed for DR AI applications, it is general enough that it may be useful in the description of any image-based diagnostic system. DR AI intended for clinical use should be in alignment with the scientific state of the art, and based on functional aspects of the system.

The characteristics of a DR AI system are interrelated:

- The *environment* may be one or more of primary care clinics, endocrinology clinics, diabetes and family care clinics, telemedicine programs, reading centers, retail walk-in clinics, ophthalmology clinics, optometry clinics, retinal specialist clinics, patient homes, and other settings.
- The *type of output* maps generally to the validation categories defined in the parent document of this appendix (ATA Telehealth Practice Guidelines for Diabetic Retinopathy); a fifth category for more comprehensive diagnosis of retinal disease in addition to DR was added^114–117^ Specifically:
- ○ A DR AI program that allows identification of patients who have no or minimal DR versus those who have more than minimal DR could be considered ATA category 1.^118–121^
- ○ A DR AI program that allows identification of patients who do not have STDR versus those who have potentially STDR could be considered ATA category 2.^114,121^
- ○ A DR AI program that allows identification of defined clinical levels of NPDR (mild, moderate, or severe), PDR (early and high risk), and DME (according to a clinical grading scheme,^122^ typically the ETDRS^123^) with accuracy sufficient to determine appropriate follow-up and treatment strategies could be considered ATA category 3.^121^
- ○ A DR AI system that matches or exceeds the ability of ETDRS photographs to identify all lesions of DR to determine precise levels of DR and DME^123^ could be considered ATA category 4.^121^
- ○ A DR AI system that can exclude or describe the presence of non-DR diagnoses, such as, but not limited to, retinal vein occlusions, hypertensive retinopathy, choroidal nevus, and macular degeneration is not currently described in the ATA categories, although the ETDRS system includes level 12 to describe non-DR findings.^117^
- The *end user* can be physicians and other providers, nonphysician staff, or patients (in a direct-to-consumer paradigm).
- Additional characteristics of intended use that can be specified are:
- ○ A specific image quality taxonomy and level required by the DR AI system.
- ○ A specific imaging protocol required by the DR AI system, which may include requirements for the size, number, and localization of fields per eye.
- ○ An ability of the DR AI system to evaluate differences in disease features between two or more visits, such as changes in lesion distribution, extent, or other characteristics representative of activity.^124^

These additional characterizations may be helpful for a full description of the capabilities and limitations of the DR AI system's intended use.

Within an intended use case, a DR AI system output characteristic should match the end user and environment characteristic. For example, a patient will typically be unable to interpret specific disease severity levels, and thus the output, that is, report, for this use case is required to include a referral or no referral result. Likewise, some physician users may have background in DR and so inclusion of specific clinical or even ETDRS classification levels may be more appropriate.

### Diagnostic Accuracy Evidence

The purpose of this section is to describe standardized levels of diagnostic accuracy of DR AI systems. This system does not specify the requirements to achieve a particular autonomy level, but rather defines the criteria by which diagnostic accuracy evidence can be evaluated by physician and patients as well as consumers and policy makers. The characterizations are descriptive and are in alignment with the current scientific state of the art, allow a step-wise progression, are based on functional aspects of the system, and define the intended use and provider roles for each level. Although the requirements for the evidence vary with the level of desired system autonomy, with a more autonomous system requiring greater scrutiny, these guidelines remain descriptive.

#### Current Good Manufacturing Practices

Current Good Manufacturing Practices are regulations enforced by the FDA to facilitate proper design, monitoring, and control of manufacturing processes and facilities.^125^ Similar requirements exist in other countries. These regulations imply that the design and production of the DR AI system are under some form of structured quality control that requires validation. For example, 21 CFR 820^126^ in the United States and ISO 13485^127^ in the European Union set forth the minimum requirements of a quality management system, including a framework for the design, development, and production of medical devices, and postmarketing surveillance.

#### Accuracy Study

A diagnostic accuracy study examines diagnostic accuracy of the DR AI system in isolation, that is, without full reflection of its intended use. Diagnostic accuracy studies for DR AI will involve images of subjects demonstrating a full range of DR and DME severity. Retinal imaging equipment operator performance, image quality management, and other factors external to DR AI systems are outside the scope of this AI discussion. Reference standards and metrics are discussed hereunder.

#### System Validation in Context

Validation as a system implies that the diagnostic accuracy of the DR AI system is examined within the entirety of its intended use (end-to-end). All factors that will affect the quality and availability of the subject's images are considered. Thus, the overall system validity will depend not only on the diagnostic accuracy of the DR AI system in isolation, but also on a variety of related programmatic components, such as the ability of a real-world operator to demonstrate technical proficiency and acquire retinal images according to the required imaging protocol, and with sufficient quality for the successful disposition of the subject.

#### Metrics for Diagnostic Accuracy and Validation Studies

Diagnostic accuracy and system validation studies should yield data relevant for management decisions of patients based on the intended use. Although variable thresholds are possible, clinical practice will require management decisions on patients to be made with fixed preset thresholds (e.g., disease is present or absent, a specific risk level is present or absent). Thus, the classical diagnostic accuracy metrics of sensitivity and specificity, which are appropriately used for binary outcomes, are more appropriate measures than metrics such as receiver operating characteristics (ROC) analysis. Similarly, they are also more appropriate than aggregate accuracy expressing a single metric, that is, combining sensitivity and specificity. In addition, newer metrics, such as severity-weighted sensitivity, incorporate the clinical significance of false negatives at different severity levels of disease (i.e., higher risk of vision loss if a case of severe DR is classified as normal as compared with a case of mild DR).^128^

Standards for diagnostic accuracy studies, such as Standards for Reporting of Diagnostic Accuracy Studies,^129^ can help in comparing DR AI systems and in increasing acceptance by clinicians and the public. In addition to the classical diagnostic accuracy metrics already described, the following are important to define the DR AI system at their specified diagnostic accuracy evidence level^112^:

- The fraction of subjects who can be successfully imaged and result in a disposition by the DR AI system, referred to as *gradability.*
- Corrected measures of sensitivity and specificity taking into consideration gradability, again with a preset threshold.
- Specific report of the severity level of DR of all false negatives.
- Use of severity-weighted metrics such as severity-weighted sensitivity.
- Evaluation of the repeatability and reproducibility of the DR AI system.
- Limit of detection of a system; that is, the robustness of the system to random and so-called adversarial inputs.^130,131^
- Analytical sensitivity reflecting how image artifacts and other disruptions affect performance.

#### Accuracy Study Setting: Laboratory or Intention to Screen

Accuracy studies can be characterized as laboratory or intention to screen.

Laboratory studies are characterized by the use of retrospectively accessed pre-existing image data sets, which may be publicly available. Typically, these data sets are enhanced by removal of low-quality images.

Intention-to-screen studies in contrast include all images to better replicate real-world conditions in which media opacity and poor dilation may preclude perfect quality photography. Such studies are characterized by either prospectively collected or previously collected under a prespecified but unrelated protocol. The data sets may include clinicaltrials.gov registration trials or image data sets that were not previously available, even though they are accessed retrospectively as long as poor image quality was not an exclusion criterion for the study.

#### Reference Standard Truth Derivation

The reference standard for a diagnostic accuracy or system validation study is typically derived from subjective reading of retinal images, but can also be derived from more objective sources, such as definitive retinal thickening by OCT or even clinical outcome.^132^ The following levels of subjective grading may be used:

Level A reference standard: A reference standard that either is a clinical outcome or an outcome that has been validated to be equivalent to patient-level outcome (i.e., a surrogate for a specific patient outcome). This reference standard is derived from an independent reading center (where the clinicians or experts performing the reading are not otherwise involved in performing the study), with validated published protocols, and with published reproducibility and repeatability metrics. A level A reference standard is based on at least as many modalities as the test and ideally more.
Level B reference standard: A reference standard derived from an independent reading center with validated published reading protocols, and with published reproducibility and repeatability metrics. A level B reference standard has not been validated to correlate with a patient-level outcome.
Level C reference standard: A reference standard created by adjudicating or voting of multiple independent expert readers, documented to be masked, with published reproducibility and repeatability metrics. A level C reference standard has not been derived from an independent reading center, and has not been validated to correlate with a patient-level outcome.
Level D reference standard: All other reference standards, including single readers and nonexpert readers. A level D reference standard has not been derived from an independent reading center, and has not been validated to correlate with a patient-level outcome, and readers do not have published reproducibility and repeatability metrics.

In addition, studies can be characterized as having reference standards derived from objective measures such as clinical outcome or OCT, or a combination of the mentioned.

Diagnostic drift, where readers differ in their grading system compared with the reading center that was involved in the original foundational studies, should be taken into account.^121^

Studies can be prospective, that is, where the data are collected according to a prespecified protocol.

Studies can be preregistered, that is, where the data and the statistical analysis, hypothesis to be tested, and subject exclusion are executed according to a prespecified protocol and statistical analysis plan. Preregistration is a requirement for publication in many scientific journals^133–135^

Conflicts of interest with the organization that is involved in the development and sponsorship of the DR AI system should be taken into account.

Additional considerations to be made in classifying the quality of diagnostic accuracy evidence.

The following variables can be used to further describe the characteristics of the diagnostic accuracy evidence for a DR AI system:

- Inclusion or exclusion of non-DR incidental findings (e.g., age-related macular degeneration, central retinal vein occlusion, and hypertensive retinopathy) in the accuracy analyses. If included, were they considered positive, negative, or both.^136^
- Selection of the grading system used to create the reference standard against which the DR AI system is evaluated, such as National Health Service United Kingdom^137^ Eurodiab^138^ or ETDRS.^123^ The choice of grading system will also depend on the intended use. The grading system affects the estimated diagnostic accuracy and performance of the DR AI system, even within the same reading center.^122,133^

#### DR AI System Design

DR AI system design has changed considerably over the past 10 years, and significant continued evolution is expected; therefore, any characterization of design must be descriptive to avoid rapid obsolescence. Nevertheless, there are some characteristics of system design that are considered informative at the present time, for example, amount of training required and explanation generation of the DR AI system.

DR AI systems can be characterized by the amount of training required. One taxonomy involves the so-called unsupervised and supervised categories.^117^ *Unsupervised* implies that once the algorithm has been designed and implemented, none of the parameters are ever adjusted in response to the performance on a training set of images. Almost all current DR AI systems are *supervised*, in which some or many parameters are adjusted during a so-called training phase in response to a specific performance on a training data set until an acceptable performance is achieved. Another category is *semisupervised*, which combines aspects of both aforementioned types to improve complex image analysis. These terms are not very useful to categorize DR AI systems.

*Explainability* (explanation generation) of a DR AI system design means that human users can understand, at least to some level of abstraction, how the DR AI system arrives at its diagnostic output (i.e., “The computer finds all the microaneurysm, hemorrhages, and exudates. Based on the total number and location of each, the final diagnosis is calculated”). Explainability is predicated upon an appreciation of contemporary DR AI system design.

DR AI system designs can use retinal feature detectors to determine the presence of lesions and biomarkers in retinal images (hemorrhages, exudates, etc.), as well as nonlinear transformations of their outputs.^139–142^ Machine learning approaches are typically used for the generation of the final output (normal vs. abnormal, etc.). Because these DR AI systems involve multiple feature detectors for pathognomonic DR lesions, they are categorized as *lesion based*, and can be explained at the disease characteristic level because they explicitly detect types of relevant lesions. Some have claimed that lesion-based designs are more “physiologically plausible”^143^ with multiple redundant lesion-specific detectors, and a functional method that mimics the human visual cortex.^144^

DR AI system designs can also involve one or more multilayer neural networks, such as convolutional neural networks (CNNs).^145^ Such designs have allowed marked improvements in diagnostic accuracy, as evidenced in the diagnostic accuracy of algorithms in the recent Kaggle competition^146^ These designs have one or more CNN trained to associate an entire retinal image with a disease-level diagnostic output. In these designs, the computer is “fed” each image and its corresponding output in a very large training set and then develops a system to grade the images without “knowledge” that microaneurysms, hemorrhages, and exudates are the hallmarks of DR, but instead uses the raw pixel data to “learn” what DR is and is not. Because the system has not been “taught” about the lesions of DR and is not explicitly using them to make the DR diagnosis, the human user cannot understand how the AI system actually makes the diagnosis of DR, and so such systems are sometimes considered *black box* designs.

A number of end-to-end-based DR AI systems have been developed in academic and other prototype contexts in recent years, leveraging the fact that extensive feature design is not required.^32,117,147,148^ Although these systems may actually detect some lesions (i.e., the system “teaches itself” about microaneurysms), the operation of *black box* systems cannot be verified or explained at the disease characteristic level.

Explanation generation has not been shown to affect overall diagnostic accuracy based on classical diagnostic accuracy metrics to date.^32,121,147^ Some claim that a lack of explanation generation may impact diagnostic accuracy and the risk of unanticipated errors from small perturbations that can be estimated with non-Gaussian diagnostic accuracy metrics.^130,131^

### Summary

In summary, this document puts forth these standardized descriptors to form a means to categorize systems for computer-assisted and fully automated detection, triage, and diagnosis of DR. The components of the categorization system include *Level of Device Autonomy*, *Intended Use*, *Level of Evidence for Diagnostic Accuracy*, and *System Design*. There is currently no empirical basis to assert that certain combinations of autonomy, accuracy, or intended use are better or more appropriate than any other. Therefore, at the current stage of development of this document, we have been descriptive rather than prescriptive, and we treat the different categorizations as independent and organized along multiple axes.

### Disclosure Statement

M.D.A. is founder, CEO, director of IDx (Coralville, IA), and an investor. M.D.A. has patent and patent applications assigned to the University of Iowa, to the Department of Veterans Affairs, and to IDx. T.L. is consultant for Spect, Inc. (San Francisco, CA). K.R. is chief medical officer of IBM, Inc. (Yorktown, NY). M.F.C. is an unpaid member of the Scientific Advisory Board for Clarity Medical Systems (Pleasanton, CA) and a consultant for Novartis (Basel, Switzerland). M.B.H. has no competing financial interests.

### Funding Information

M.D.A. is the Robert C. Watzke Professor of Ophthalmology and Visual Sciences, and supported by NIH grants R01 EY019112, R01 EY018853; by unrestricted departmental funding from Research to Prevent Blindness (New York, NY), the Department of Veterans Affairs, and Alimera Life Sciences. T.L. is supported by unrestricted departmental funding from Research to Prevent Blindness, and in part by the Heed Ophthalmic Foundation Fellows Grant. M.F.C. is supported by grant P30EY010572 from the National Institutes of Health (Bethesda, MD), and by unrestricted departmental funding from Research to Prevent Blindness.

### Appendix A4. Health Insurance Portability and Accountability Act

The HIPAA of 1996 established privacy protection for individually identifiable health information. State laws may offer additional protection for specific types of health information.

Key privacy elements include:

- Covered entity: any organization, person, or business associate thereof that transmits PHI in electronic or paper form.^149^
- Covered information: individually identifiable health information in any form or medium used or disclosed by a covered entity.^150^
- Voluntary consent: covered entities may obtain a patient's consent before using or disclosing his or her health information.

The HIPAA security standards for the protection of electronic PHI protect the confidentiality, integrity, and availability of covered information by covered entities through technical (software and hardware) and nontechnical (policies) means that are reasonable and scalable. The “security rules” do not apply to nonelectronic format of personal health information. Key electronic PHI security includes:

- Confidentiality: requires authentication and role-based access to data (must have a need to know).
- Integrity: requires methods for assuring no unauthorized altering or destruction of data.
- Availability: requires methods for disaster recovery (e.g., fire, vandalism, system failure, and natural disaster), backup, and access to data under all conditions.^151^
- Security management: policies and procedures to prevent, detect, contain, and correct security violations^152^; periodic evaluation of security management.
- Security awareness and training for all program staff.

### Appendix A5. Privileging and Credentialing

CMS, TJC, and other health care accrediting bodies provide standards for privileging and credentialing providers. Historically, these standards placed undue burden on telemedicine programs without improvement in quality of care or provider accountability. This burden has been managed variably for telemedicine providers since TJC provided privileging and credentialing by proxy for telemedicine providers in 2003. In 2011, CMS adopted similar but expanded rules that allow a Medicare-participating facility receiving telemedicine services (OS) to rely upon the credentialing process and privileging decision for the telemedicine providers of the accredited health care facility providing those telemedicine services (DS). CMS defined this process in its Final Rule (76 FR 25550).^153^ These regulations were reflected in TJC Medical Staff and Leadership standards (MS.13.01.01, MS.10.01.03, and LD.04.03.09).^154^ The key requirements of these regulations and standards include:

1. A written agreement is in place between the OS and DS wherein the DS ensures to meet or exceed all applicable Medicare conditions of participation (CoP) and standards required of the OS regarding privileging and credentialing the telemedicine provider(s).
2. The DS is a Medicare-participating hospital or critical access hospital (CAH), or telemedicine entity. Telemedicine entity is not statutorily defined but was created by CMS in the final rule to further specialty service access and reduce administrative burden while preserving patient safety. A telemedicine entity acts as a DS telemedicine service and has the following features:
3. a. Provides telemedicine services.
4. b. Is not a Medicare-participating hospital (therefore, a non-Medicare-participating hospital that provides telemedicine services would be considered a DS telemedicine entity also).
5. c. Provides contracted services in a manner that enables a hospital or CAH using its services to meet all applicable CoPs, particularly those requirements related to the credentialing and privileging of practitioners providing telemedicine service.
3. The DS telemedicine provider is privileged at the DS hospital for the same services to be provided at the OS.
4. The DS hospital provides a current list of the telemedicine provider's privileges.
5. The DS hospital conducts an internal review (ongoing professional practice evaluation) and provides evidence of this review to the OS.
6. The DS must make a recommendation to the OS to provide telemedicine services.
7. The organized medical staff at the OS must make a recommendation to the OS's governing body.
8. The OS hospital has an internal review of the DS telemedicine provider's performance and provides this information to the DS for its evidenced-based process of recredentialing. This must include:
12. a. All adverse events (AEs) relating to the DS provider.
13. b. All complaints about services from the telemedicine provider.

### Appendix A6. Quality Control

Quality control and performance improvement must address the entire program. Each quality measure should be specific, quantifiable, achievable, realistic, time bound, evidenced based, and tailored to the program's objective.^98^ The safety and effectiveness of a telemedicine program for DR are largely established by its validation studies. However, a rigorous and ongoing quality control program is needed to ensure that the clinical operations maintain high fidelity with the validation studies over time and across all deployment sites. The following are major categories of performance that **should** be evaluated by most programs, although some may not be applicable in all cases:

- OS
- ○ Administrative
- ▪ Primary care provider and nursing satisfaction surveys
- ▪ Patient satisfaction surveys
- ▪ DR surveillance rate for catchment area of the program
- ▪ Successful patient enrollment rate (sustained vs. initial)
- ▪ Successful referral completion rate and timeliness.
- ○ Imager
- ▪ Ungradable study rate
- - Retinal field definition
- - Image focus
- - Stereo pair separation and alignment.
- ▪ Imaging time
- ▪ CE.
- ○ Equipment
- ▪ Preventative maintenance schedule.
- Reading center
- ○ Administrative
- ▪ Average acquisition to reader time
- ▪ Average reading time: routine cases
- ▪ Average reading time: stat studies
- ▪ Average acquisition to report delivery time
- ▪ Exception rate and time (variance from program goals).
- ○ Technical: network, servers, software, etc.
- ▪ Connectivity losses: number, duration
- ▪ Servers: nonnetwork-related offline events
- ▪ Software: known bugs, new bugs, duration.
- ○ Reader
- ▪ Average reading time
- ▪ Peer review outcome.
- - Adjudicator agreement
- - External review
- - Test set performance.
- ▪ Ungradable rate
- ▪ Agreement with live examination.
- - Random sampling
- - Referrals.
- ▪ CE
- - Completions
- - Timeliness.

Multiple feedback loops in a quality control program allow CE programs to identify trends and adapt to changing conditions. These reviews allow CE performance and cost-effectiveness to be continuously enhanced (*Fig. 3*), resulting in a process of continuous quality improvement (CQI).

The following are examples of training linked to a QA protocol:

- Standardized training for imager, imager trainers, readers, and reader trainers.^140^
- Structured, self-study, pretraining of imager and reader to provide baseline background knowledge.
- Structured curriculum with defined endpoints and criteria-based demonstrated proficiency.
- Provisional certification followed by full certification based on experience with a minimum number of telemedicine studies over a defined period of time. Experience should demonstrate required levels of proficiency documented by quality review of a fixed number of cases.
- Time-limited certification of imagers and readers. Recertification should be based on the period since last clinical encounter (recency), number of clinical encounters over a period of time (currency), and proficiency as documented by formal review. Ocular telehealth programs should create certification methods that are defined and relevant to the program.

Ongoing sampling of imager and reader performance by criteria-based review should be performed with a periodicity that satisfies local policy and regulatory requirements for the OS and DS. A review of trends in fallout from outcome analyses can be used to assess:

- Proficiency.
- Opportunities for program improvement.
- Need for changes in initial or recurring training.
- Need for additional training of an imager or reader.
- Evidenced-based reprivileging of licensed readers.

CE is an important component of any QA/CQI program and a key method for ensuring current competency.^141^ CE should be dynamic and sensitive to patient and staff's changing needs. The following are considerations in selecting specific CE:

- Adjust CE content by end-to-end program testing through data sampling and outcome analysis.
- Adjust CE program to maintain relevance to the specific population served by the program.
- Deliver CE in formats to achieve desired outcome with maximum efficiency and effectiveness. Format examples include periodic self-study curriculum with pre- and poststudy testing, newsletters, and e-mail “Tips of the Day.” A variety of interactive CE sessions using telehealth technology are available, such as group-based or one-on-one case reviews, morbidity and mortality conferences, and conferences patterned on clinical pathological conference concepts.

Similar guidance comes from broadly distributed programs outside the United States. The U.K. National Screening Committee adopted digital photography in 2000 for a systematic national risk reduction program.^16^ Their model incorporates trained professionals, recorded outcomes, targets and standards, QA, and promotion to increase screening rates. Criteria and minimal/achievable standards were proposed for each QA objective.^155^ Other ongoing QA programs are publishing measures and outcomes. For example, a U.K. diabetes center regraded a percentage of images to determine appropriateness of referrals for clinic examination.^156^ Other programs have reported outcomes measuring image quality, intragrader reliability, and percentage of grader-generated reports within 48 h of grading images.^157^

### Appendix A7. Customer Support

Telemedicine programs will require ongoing support to remedy hardware and software malfunctions. Programs **may** find it appropriate to consider the following example of an ocular telehealth program three-level help desk.

### Level 1

This is the entry point for most/all initial support requests. Support staff can satisfy routine image acquisition issues and entry-level troubleshooting of software and data transmission. If the request for support is determined to be outside level 1 scope, the call is triaged to the level 2 or level 3 help desk.

### Level 2

For more complex software and data transmission issues, a second level support is needed to provide solutions that are more technically complex, but not requiring software or network engineer intervention. The level 2 support staff sometimes is a bridge between levels 2 and 3 services. This is a function that typically evolves over time as the level 2 staff becomes more experienced with the operational idiosyncrasies of the technology.

### Level 3

A third level support is needed for troubleshooting and resolving proprietary technology or particularly complex network issues. This support usually involves the imaging device and associated technology, and diagnostic software (camera backs, relay lenses, imaging and reading software applications, etc.).

Help desk response expectations **should** be prioritized to the program impact level of the exception raising the request for support. The following table provides examples of impact-level stratification and resolution timelines (*Table 5*).

**Table 5.** Help Desk Impact Level Stratification and Resolution Timelines

IMPACT LEVEL	DEFINITION	TARGET CALL BACK TIME	TARGET RESOLUTION TIME
1	Critical:	15 min	4 h
	Critical system software is entirely unavailable or severely degraded to the point of unusability and there is no workaround/alternative		
2	Major:	1 h	8 h
	Noncritical system software is entirely unavailable or;		
	Critical system software is entirely unavailable or severely degraded to the point of unusability and there is a workaround/alternative		
3	Minor:	1 business day	14 business days
	Part of a system is unavailable		
4	Nonurgent user interface issues:	1 business day	21 working days
	System has failed to meet its specification		
	or;		
	Request for information about how to use the system		
5	Good will:	1 business week	90 days
	Anything else; e.g.		
	• State changes		
	• Letter changes		
	• *Patient merges*		
	• Resetting grading		
	• Correcting observations		

### Appendix A8. Reimbursement

The reimbursement landscape is highly dynamic, and has substantial state, regional, and payer differences. Failure to attend to these changing differences appropriately can result in failed reimbursement and in some instances, costly penalties. For these reasons, programs **should** seek expert council to ensure compliance with the requirements of a particular payer and locale. Telehealth Resource Centers (TRCs) are an excellent source of regional-specific information on reimbursement issues. (www.telehealthresourcecenter.org/reimbursement) At the time of publication, there was active legislative debate pertaining to expansion of Tmed-DR reimbursement, with emphasis on CPT codes and protocols. Programs **should** contact their regional TRC for current specifics on DR ocular telehealth reimbursement in their state or region (www.telehealthresourcecenter.org/who-your-trc).

CPT^158^ provides several category 1 codes that can be used by telemedicine programs for DR screening or diagnosis (Tmed-DR). The specific code(s) for a particular program depends upon the state, regional, payer, and program characteristics. The following CPT codes are available within these constraints.

### Medicare

CPT 92227: remote imaging for detection of retinal disease (e.g., retinopathy in a patient with diabetes) with analysis and report under physician supervision, unilateral or bilateral.

CPT 92228: remote imaging for monitoring and management of active retinal disease (e.g., DR) with physician review, interpretation and report, and unilateral or bilateral.

These remote retinal imaging codes, introduced in 2011, allow for detection of retinal disease (92227) and the monitoring and management of active retinal disease (92228). The codes specifically address the clinical application of telemedicine modalities for DR. The providers who might use these codes are primary care providers, imaging centers, optometrists, and ophthalmologists.

The primary differences between the codes are based upon professional interpretation of the images, and presence of clinically evident DR. CPT 92227 does not require a professional interpretation, whereas CPT 92228 does. CPT 92227 describes a screening service provided by a technician with physician supervision (level of supervision not specified), which may or may not identify retinal disease, whereas CPT 92228 is an assessment of existing retinal disease for remote management. Since CPT 92228 requires interpretation by a licensed independent provider (LIP), it is a greater service than 92227 in both scope and value. Medicare covers CPT 92228 because this service is performed “… for the diagnosis or treatment of illness or injury or to improve the functioning of a malformed body member.”^159^

However, screening services are usually considered to be noncovered in the absence of a statutory provision to the contrary (e.g., glaucoma screening). Several Medicare Administrative Contractors (MACs) have published local coverage determinations on this topic, so programs **should** check their MACs for further details.

These codes have been problematic since their inception, since they poorly define the role of Tmed-DR, do not satisfy the most common telemedicine use cases for DR, and undervalued services provided by DR telemedicine applications.^160^

In its protest, the ATA noted that the CPT 92227 definition does not reflect the large majority of actual DR remote retinal imaging programs, and that CPT 92228 does not reflect the complexity of care associated with DR remote imaging. Since CPT 92227 applies to programs with technical (non-LIP) readings, it assigns zero relative value units (RVUs) to physicians' work. CPT 92228 significantly undervalues the physician's responsibility and care. ATA also expressed concern that CPT 92228 restricts reimbursement to only patients with active retinal disease. Importantly, this prevents qualifying programs from reimbursement for population surveillance since the presence of retinopathy in new patients cannot be predetermined without a retinal examination. Total RVUs assigned to these new codes are markedly less than the previously used CPT 92250 (fundus photography), although similar equipment, staff, and physician effort are involved.

Currently, CPT 92227 and CPT 92228 are the only dedicated telemedicine codes available for DR screening/diagnosis. Given the restriction in these CPT code definitions, one possible approach is their combined and selective use by a single program. In this use case, a program could use technology and operations satisfying CPT 92227 for annual screening of patients with DR. Those patients shown to have clinically evident DR below the referral threshold are eligible for reimbursement through a “92228” program on subsequent periodic examinations. Those patients without DR could be screened annually using the “92227” program component. The concerns raised by the ATA have not yet been addressed.

### Medicaid

Coverage, coding, and valuation of Tmed-DR are state dependent. Programs **should** contact their state Medicaid office to determine Tmed-DR coverage, appropriate codes and comments, and other billing particulars for their program. In general, the reimbursement for Medicaid is 10–20% lower than the same service covered by Medicare.

### Commercial Insurance Carrier

Many private and commercial carriers reimburse DR Tmed-DR using CPT code 92250 (Fundus Photography with interpretation and report), 92227, and 92228. Some allow use of the level II HCPCS code, S0625 (Retinal Telescreening by Digital Imaging of Multiple Different Fundus Areas to Screen for Vision-Threatening Conditions). Some carriers reimburse for the service but require pupil dilation. Owing to this variation among carriers, each must be contacted to determine the requirements for reimbursement.

Tmed-DR may be reimbursed as a single complete service, or fractionated into defined components. A global procedure contains both professional and technical components. Suffixes applied to certain Tmed-DR CPT codes provide for splitting reimbursement to separate providers based upon the performed component.

- 26: The professional component represents the supervision and interpretation of a procedure provided by the physician or other health care professional. It is identified by appending modifier *26* to the procedure code.
- TC: The technical component represents the cost of the equipment, supplies, and personnel to perform the procedure. It is identified by appending modifier *TC* to the procedure code.
- A global service includes both professional and technical components. The global service is identified by reporting the eligible code without modifier 26 or TC.

### Other Financial Factors

#### Logistic Efficiencies

Geographic disparities in care can result in access to care issues that are costly in terms of time, transportation, and missed opportunity. Telemedicine can close these distances electronically with a possible overall savings in costs to the patient and health care system.

#### Disease/Complication Prevention

Increasing the surveillance rate of DR through telemedicine contributes to increased treatment and reduction in retinal complications and related vision loss.^161,162^ This reduction can result in significant health care savings through cost avoidance.^163,164^ In principle, this is the predominate business case for Tmed-DR, but the U.S. health care model limits its use in most cases since the primary care provider supporting the program is not the immediate recipient of the cost avoidance benefits. This shortcoming is somewhat offset by the trend of pay-for-performance incentives.

#### Resource Utilization

Some DR telehealth programs have shown to be less costly and more effective than conventional retinal examinations for the detection of DR.^165^ In addition, use of telehealth-DR may allow a reduction in the overall cost of care with the same or expanded scope of services through the retasking of costly human resources. However, depending on the structure and business model of health care system and reimbursement methods used, these cost savings may not provide benefit to all providers and programs participating in the telehealth-DR service.^21^

### Appendix A9. Telemedicine for Glaucoma: Guidelines and Recommendations

Kenman Gan, MD,^1,2^ Yao Liu, MD, MS,^3^ Brian Stagg, MD, MS,^4^ Siddarth Rathi, MD, MBA,^5^ Louis R. Pasquale, MD,^6^ and Karim Damji, MD, MBA^1^

^1^Department of Ophthamology and Visual Sciences, Faculty of Medicine and Dentistry, University of Alberta, Edmonton, Canada.

^2^Department of Ophthalmology and Visual Sciences, Faculty of Medicine, University of British Columbia, Vancouver, Canada.

^3^Department of Ophthalmology and Visual Sciences, University of Wisconsin-Madison, Madison, Wisconsin.

^4^John Moran Eye Center, University of Utah, Salt Lake City, Utah.

^5^NYU Langone Health, New York, New York.

^6^Department of Ophthalmology, Icahn School of Medicine at Mount Sinai, New York, New York.

### Background

Glaucoma is the leading cause of irreversible blindness worldwide, estimated to affect >60 million people.^166^ This condition is clinically defined as a group of progressive optic neuropathies having characteristic patterns of visual field loss. In the United States, the prevalence of glaucoma ranges from 2% among those aged 40–49 years and increases with age to 8% or more among those aged 80 years or older.^167^ The estimated yearly direct cost for glaucoma management in the United States exceeds $2.9 billion.^168^

Current diagnostic and treatment guidelines for glaucoma are informed by several large multicenter clinical trials.^169,170^ Glaucoma care guidelines are not as standardized as those for DR, which allow for significant regional and provider variability in glaucoma diagnosis and management.^170^ It is important to note that other areas of medicine—including psychiatry and primary care—have flexible practice guidelines, which have not been a barrier to the successful large-scale uptake of telemedicine in these fields.

### Introduction

Teleglaucoma is a growing field with great promise for increasing patient access to high-quality cost-effective glaucoma care by leveraging new telecommunications and diagnostic technologies. Many of the telehealth principles underlying teleglaucoma programs are shared with those in teleretinal programs for DR. In this appendix, we review some additional considerations and practice recommendations for teleglaucoma programs.

### Discussion

#### Definition and Literature Review

Glaucoma is a chronic lifelong disease for which patients are monitored at clinic visits occurring one or more times yearly. Access to glaucoma specialists is becoming more limited as the prevalence of glaucoma is expected to increase dramatically with our aging populations.^166,171^ Advances in telecommunications and diagnostic technologies have allowed for the development of teleglaucoma programs, wherein key glaucoma measures are collected from a patient at an OS and then transmitted to a DS provider for interpretation. Teleglaucoma has the potential to increase access to glaucoma care by improving efficiency and decreasing the need for long-distance travel for patients.^172^ There is an emerging body of literature to support teleglaucoma programs.^173–187^ In addition to these published reports, there are also many active clinical teleglaucoma programs.

Kotecha et al. reported using teleglaucoma to decrease the amount of time patients spent during each clinic visit.^188^ Another study found that approximately three-quarters of glaucoma suspects evaluated remotely did not require in-person follow-up examination.^186^ A cost-effectiveness analysis found that teleglaucoma was more cost-effective than in-person examination for glaucoma screening.^189^ Patients participating in teleglaucoma programs report comparable satisfaction with in-person examinations.^190^ A number of teleretinal programs have published high rates of incidental detection of glaucomatous-appearing optic nerves and suspected glaucoma is a major contributor to clinical referrals in teleretinal diabetic screening.^191,192^

#### Types of Teleglaucoma Programs

The extent to which a given teleglaucoma program can support various types of use cases depends greatly on the resources available as well as the training and comfort level of the providers. We define a “Full Scope” teleglaucoma program as one with sufficient resources to provide not only glaucoma screening but also diagnosis and treatment monitoring. The types of teleglaucoma programs can be described on the following spectrum of use cases.

##### Screening

Screening for glaucoma refers to the systematic evaluation of asymptomatic persons for evidence of glaucomatous damage.^193^ In 2013, the U.S. Preventive Services Task Force concluded “the current evidence is insufficient to assess the balance of benefits and harms of screening for primary open-angle glaucoma in adults.”^194^ However, subsequent studies have suggested that screening in populations at high risk for glaucoma is effective.^195,196^ A systematic review of teleglaucoma screening estimated its sensitivity as 83.2% and specificity as 79.0%.^193^

##### Diagnostic consultation

Teleglaucoma can also be used to provide specialist consultation from a distance to reduce patient travel.^186,197^ Verma and coauthors reported that 69% of teleglaucoma patients referred for suspected glaucoma could be managed by the referring primary eye care provider and did not require in-person evaluation by a specialist.^186^

##### Long-term treatment monitoring

Teleglaucoma can also be used for follow-up monitoring after initiation of treatment. “Virtual Glaucoma Clinics” described in the United Kingdom use teleglaucoma for long-term treatment monitoring.^174,187,188^ In these clinics, “stable” patients are followed through virtual review of glaucoma testing with in-person visits only when necessary.^188^ Kashiwagi et al. have described a slit-lamp camera system to accurately monitor delegation of postoperative care to nonglaucoma specialists after glaucoma surgery.^198^

#### Key Components of Teleglaucoma Programs

Additional key components in establishing teleglaucoma programs include the following.

##### Patient history

Important components of the patient history include any ocular and visual symptoms, demographics, ocular history (including the use of any eye medications, last eye examination and recommended follow-up, and previous diagnosis of DR), medical history, and family history (e.g., first-degree relatives with glaucoma and the severity of their disease).

##### Equipment

Equipment needs for each teleglaucoma program depend on the program's goals, provider preferences, patient population, and the availability of community resources. Some important components may include:

1. Visual acuity testing.
2. Visual fields. Reliable visual field testing is required for (1) establishing baseline visual fields for future comparisons and (2) detecting progressive visual field loss in patients with worsening glaucoma.^170^ Automated static threshold perimetry with white-on-white stimuli is considered the gold standard for diagnosis and monitoring of glaucoma.^170^ The Swedish interactive thresholding algorithm is a commonly used testing algorithm. Frequency-doubling technology and short-wavelength automated perimetry may be useful in detecting early disease, and their use in a teleglaucoma program can be considered.^199^ Two commonly used machines for automated perimetry are the Humphrey Field Analyzer (Carl Zeiss Meditec, Jena, Germany) and the Octopus Perimeter (Haag-Streit, Koniz, Switzerland).^200^ In the future, remote testing using web- or tablet-based programs may be useful.^201,202^
3. Intraocular pressure. Multiple devices are available for measuring intraocular pressure. Some devices, such as the iCare tonometer, (iCare USA, Raleigh, NC) do not require instillation of a topical anesthetic and may even be used by patients at home. (iCare HOME; iCare USA).^203,204^ Continuous intraocular pressure monitoring systems may play a role in future teleglaucoma programs (SENSIMED Triggerfish contact lens sensor, Sensimed AG, Lausanne, Switzerland).^205,206^ However, applanation with a topical anesthetic is still considered the gold standard for intraocular pressure measurement.^170^
4. Pachymetry. Central corneal thickness has become an important measure for evaluating glaucoma risk and for setting individualized intraocular pressure goals.^170,207^ Options for measuring central corneal thickness include ultrasound, low-coherence reflectometry, and Scheimpflug photography.^208^
5. Anterior chamber imaging/gonioscopy. Devices that image the anterior chamber, such as anterior segment OCT and Scheimpflug photography, can assist in identifying patients at risk for narrow angle glaucoma, but the precise threshold for treatment with peripheral iridotomy has yet to be widely agreed upon.^209^ The standard-of-care for the evaluation of anatomic narrow angles and angle-closure glaucoma remains in-person assessment of the angle by a provider using traditional gonioscopy. Remote operating slit-lamp microscopes may play a more important role in the future, as these could allow for improved diagnosis of secondary glaucomas.^198^
6. Fundus photography. Fundus photography allows providers to qualitatively assess the optic nerve. The correlation between stereoscopic (three-dimensional) fundus photographs and ophthalmologist optic disk assessment has been well validated.^210^ Studies suggest that three-dimensional photographs are superior to two-dimensional photographs for glaucoma evaluation.^211^ However, further research is needed to determine the relative benefits of these two modalities in meeting the clinical needs of teleglaucoma programs. The additional flash photography needed to obtain stereo images may lead to a reduction in the patient's pupil size and lead to inadequate image quality in nonmydriatic photography.
7. Retinal nerve fiber layer (RNFL) imaging. The RNFL thickness adjacent to the optic nerve is a commonly used objective measure for monitoring possible glaucoma progression.^212^ Measurement of the RNFL thickness has also been used for glaucoma screening.^213,214^ The data are most useful when compared with age-matched controls. However, there are significant artifacts and anomalies that can produce false-positive results, particularly in patients with high degrees of nearsightedness.^215^ Examples of equipment used to measure the RNFL thickness include OCT and Heidelberg retinal tomography.
8. Additional equipment. Tests for refractive error and color vision may also be helpful.

##### Software

Clinical decision-making for glaucoma requires the complex synthesis of a variety of measures over time. Thus, it is important for software to enable time-efficient clinical workflows. Several companies offer glaucoma-focused software programs that allow providers to rapidly evaluate multiple components of longitudinal patient data, often viewed concurrently within a single screen (e.g., Zeiss Forum; Carl Zeiss Meditec and Care1 Telemedicine Network, British Columbia, Canada). AI software for image analysis may play a role in the future of teleglaucoma.^216^

##### Personnel

Skilled providers, technical, and administrative staff support are needed for both collecting and reviewing the complex data needed for teleglaucoma programs. The personnel and/or providers at the OS(s) play an important role in ensuring high-quality data collection, detailed patient history taking, and, in some cases, appropriate patient education and counseling. Counseling topics can range from providing general information about glaucoma diagnosis to detailed discussions regarding medication adherence, the assessment of medication-related side effects, and the risks and benefits of various treatment options. Interdisciplinary collaborations between providers at the OS and DS can be beneficial in providing a high level of teleglaucoma care.^186,197^

Providers at the DS can perform consultations either in real time or using a store-and-forward model. Either option may be acceptable with sufficiently detailed documentation and instructions communicated to the patient and personnel at the OS. Personnel involved in administrative and information technology support are often closely linked with the DS/central site. A dedicated program coordinator can be invaluable for ensuring the smooth operation of teleglaucoma programs.

##### Financial

Start-up and ongoing maintenance costs associated with teleglaucoma programs are generally much higher than those of teleretinal programs due to extensive equipment, software, and personnel requirements already described. A recent systematic review found that the mean reported cost of establishing a teleglaucoma screening program ranged from $89,703 to $123,164.^193^ Although teleglaucoma screening requires substantial resources, a follow-up study estimated that using telemedicine to screen for glaucoma was more cost-effective than in-person examinations, with predicted savings of $27,460 per quality-adjusted life year (QALY).^189^

Teleglaucoma programs in Canada and Australia have obtained reimbursement.^177,217^ Reimbursement to offset technical and personnel costs are important for encouraging sustained utilization of these services.^217^ Further advances in technology may make teleglaucoma programs more affordable in the future.

##### Implementation

Owing to the high equipment, personnel, and financial start-up costs associated with teleglaucoma, it may be beneficial for providers to implement a collaborative model of care. Some implementation models include:

1. *Traditional telemedicine*. A teleglaucoma program purchases all equipment and provides trained personnel for a remote site. This model is the most expensive and is more commonly implemented in teleretinal programs for DR, which may have fewer equipment and training needs.
2. *Collaborative telemedicine*. Partnerships are created between providers with access to different types of equipment, levels of glaucoma expertise, and patient access. This model may involve collaborations between various types of providers and can increase the financial feasibility of these programs.
3. *In-house telemedicine*. Providers utilize equipment and staff in their own clinic to deliver teleglaucoma care.

Special mention should be made for “Digitally Integrated Visits,” a specific implementation of *In-House Telemedicine*, wherein glaucoma patients have a subset of clinic visits reserved solely for the purpose of glaucoma testing, such as visual fields, performed by technical personnel without seeing the provider at the same visit. The provider then reviews the test results and may respond by making changes to the patient's care plan. This system reduces the number of in-person provider visits while ensuring provider oversight of the patient's care. It is important that the patient and technical personnel have the option to escalate patient care to in-person visits with the provider when requested.

### Conclusions

Teleglaucoma has tremendous potential to improve patient access to high-quality cost-effective glaucoma care. We have reviewed some special considerations needed to address the complexity of providing guideline-concordant glaucoma care. A wide spectrum of teleglaucoma implementations is currently used around the world. The growing body of literature and experience from active teleglaucoma programs will continue to inform further development of these programs. We anticipate that teleglaucoma will have an increasingly important public health impact through expanding access to the high-quality care for glaucoma patients worldwide.

### Disclosure Statement

K.G. is the Ophthalmology Director of Care 1 Inc.; L.R.P. is a consultant to Verily, Eyenovia, Bausch + Lomb, Nicox and Emerald Bioscience.

### Funding Information

Y.L. was funded in part by an institutional grant from Research to Prevent Blindness to the Department of Ophthalmology and Visual Sciences, University of Wisconsin-Madison, and by National Institutes of Health K23 EY026518.

B.S. is supported in part by an Unrestricted Grant from Research to Prevent Blindness, New York, NY, to the Department of Ophthalmology & Visual Sciences, University of Utah.

L.R.P. was funded in part by a grant from the National Eye Institute.

The content is solely the responsibility of the authors and does not necessarily represent the official views of the National Institutes of Health or Research to Prevent Blindness.

### Appendix A10. Telemedicine for Retinopathy of Prematurity

Christopher J. Brady, MD, MHS,^1^ Samantha D'Amico, MS,^2^ and J. Peter Campbell, MD, MPH^3^

^1^Division of Ophthalmology, Department of Surgery, Larner College of Medicine, University of Vermont Medical Center, Burlington, Vermont.

^2^Division of Ophthalmology, Department of Surgery, University of Vermont Medical Center, Burlington, Vermont.

^3^Casey Eye Institute, Oregon Health and Science University, Portland, Oregon.

### Introduction

Retinopathy of prematurity (ROP) is a disease of the retinal vasculature that remains a leading cause of childhood blindness in the United States and abroad.^218,219^ The disease affects the most severely premature and lowest birth weight infants, with 15.6% of premature newborns with hospital stays >28 days and 68% of infants with birth weights <1,251 g affected by the disease.^219^

Several studies have confirmed the ability of timely treatment to prevent blindness; therefore, a simple, valid, noninvasive, and inexpensive screening examination is necessary to identify infants who are at increased risk for developing ROP.^220–223^ The availability of ROP screening is a requirement for level IIIB neonatal intensive care unit (NICU) designation in the United States, which supports infants with extreme prematurity, extremely low birth weights, or severe/complex illnesses.^224^ Although a matter of some debate in the ophthalmic literature,^225,226^ the reference standard for the screening and diagnosis of ROP is a live dilated examination by an ophthalmologist using binocular indirect ophthalmoscopy (BIO), often with scleral indentation.^227^

However, in many areas, there are a limited number of ophthalmologists and significant workforce limitations, including concerns about medicolegal liability, low reimbursement, and work-flow difficulties, such that few trained ophthalmologists may be available and/or willing to perform ROP screening examinations.^228–230^

In addition, the significant variability that exists between examiners diagnosing ROP using BIO also suggests that the use of remote imaging and computer-based image analysis (CBIA) methods may improve accuracy and consistency of diagnosis of plus disease.^228,231–234^ A study evaluating ROP image grading by eight ROP experts found that there is poor agreement on the classification of plus disease, despite established international standards.^231^ This disagreement suggests that treatment recommendations likely vary among providers and that some infants may be undertreated while others are over treated for ROP.^231^

For these reasons, there has been growing interest in photographic screening and remote interpretation for ROP screening, with reports from several successful clinical implementations and research studies available in the literature. Consequently, the 2013 joint guidelines from the American Academy of Pediatrics, American Academy of Ophthalmology, American Association for Pediatric Ophthalmology and Strabismus, and the American Association of Certified Orthoptists recognized the interest in remote interpretation of retinal images and allowed for the possibility of alternative screening strategies.^235^

Current guidelines support the use of remote digital fundus imaging to identify individuals with referral-warranted ROP (RW-ROP), but recommend that at least one BIO examination is completed before initiation of treatment or termination of ROP monitoring, as current cameras do not allow for adequate view of the peripheral retina.^229,236,237^ Still, many remain skeptical of the safety and efficacy of telehealth screening as evidenced by a survey of 847 Level III NICU directors in which only 21% of NICUs used retinal imaging devices and only 30% agreed that telemedicine for ROP screening is safe.^238^

### Background

Reports from the early 21st century documented proof-of-principle for ROP telehealth screening, but raised concerns about the technical ability of the RetCam device (Natus Medical, Inc., Pleasanton, CA) to capture images with sufficient sensitivity to replace live screening.^239,240^ However, subsequent reports began to show improved diagnostic capability and low false-negative rates, which must be minimized in any ROP screening scenario given the severe consequences of even a single missed case.^241–247^

Nevertheless, debate continues and most authors conclude that wide-field imaging could potentially serve as an adjunct to live screening, but ought not replace in-person examination by an ophthalmologist.^229,245,246^ A 2008 systematic review likewise concluded “the evidence base is not sufficient to recommend that retinal imaging be routinely adopted by NICUs to identify infants who have serious retinopathy of prematurity.”^248^ Most guidelines continue to be hesitant about ROP telehealth screening and continue to recommend a hybrid approach, given that few large-scale outcome comparisons have been published.^237^

#### Recent Clinical Study Findings

The most recent large multicenter validation study to be published, the Telemedicine Approaches to Evaluating Acute-phase ROP Study (e-ROP), compared wide-field retinal imaging performed and interpreted by nonphysicians to examinations performed by physicians.^218,249^ The e-ROP Study enrolled 1,257 infants who received a median of 3 imaging sessions and conventional live examinations at 12 sites in the United States and 1 site in Canada between 2011 and 2013. The infants had a median birth weight of 860 g and a median gestational age of 26 weeks. Approximately 44% of infants were nonwhite or did not have race information available. Any ROP was identified in 63.7% of infants and RW-ROP (plus disease, ROP in zone I or stage 3 ROP or greater)^242^ was noted in 19.4% of infants on criterion-standard live examination.

When both eyes were analyzed together (i.e., at the level of the infant), remote grading by trained nonphysician graders had 90% sensitivity and 87% specificity. Given the prevalence of RW-ROP in this population, this conferred a 97.3% negative predictive value and 62.5% positive predictive value. Importantly, when considering only those infants ultimately treated for ROP, the sensitivity of remote imaging grading was 98.2%. Although this number is impressively high, in absolute terms, there were 3 infants out of the 162 treated who did not have RW-ROP detected on the remote imaging preceding treatment.

The e-ROP authors argued that their study supports the validity of using nonphysician imagers and graders for remote detection of RW-ROP, similar to how reading centers are structured for other ophthalmic conditions.^218,250^ The authors did highlight the limitations of identifying important features of ROP and inherent variability of the criterion standard live examination as a potential weakness of the study, but also note that the possibility of missing severe ROP needs to be considered in the development of any screening program.^218,251^ The e-ROP Cooperative Group found that the region of the retina where most severe disease occurs (zone I) may be best assessed by retinal images, but that the subtleties that may be seen in stage 3 ROP in zone I may currently be best identified by an experienced clinician on a live examination.^251^

The Imaging and Informatics in Retinopathy of Prematurity Consortium (i-ROP) found that there was a slightly higher accuracy for diagnosis of zone III and stage 3 ROP on live examination than with imaging.^252^ Although examinations by an experienced clinician currently remain the gold standard for ROP screening, there are weaknesses to this system to which the implementation of tele-ROP screening and automated image analysis may be the solution.

Turnaround times of 24 h or less were feasible in the e-ROP study, with >95% of images returned within that period, showing that ROP telemedicine is capable of providing timely feedback for detection of ROP.^228^ The biggest barriers to rapid turnaround identified were the time of submission and delays between image acquisition and uploading. Images that were submitted before 2 pm were graded much more quickly than images that were submitted later and, therefore, not graded until the next morning. The authors felt these issues could be addressed by improving technology used to select and submit images to allow images to easily be submitted at the bedside and increasing staffing at reading centers during peak demand times.

#### Reports of Clinical Implementations

##### United States

A hybrid model has been deployed at six NICUs in Northern California through the Stanford University Network for Diagnosis of Retinopathy of Prematurity (SUNDROP) in which all infants meeting screening criteria are photographed according to screening guidelines and then also receive a live examination within 1 week of NICU discharge. A retrospective analysis of 6 years of follow-up between 2005 and 2011 has been published.^219^ During this time, 1,216 eyes were screened, generating 26,970 retinal images. Twenty-two infants were determined to have treatment-warranted ROP (TW-ROP: zone I, any stage ROP with plus disease; zone I, stage 3 ROP with or without plus disease; zone II, stage 2 or stage 3 ROP with plus disease; any plus disease; or any stage 4 or higher disease).^221^

All TW-ROP infants were successfully identified through photoscreening in this time period and only 1 “false-positive” case was noted in which stage 3 ROP was not felt to warrant treatment on live examination. These results translate to a sensitivity of 100%, negative predictive value of 100%, specificity of 99.8%, and positive predictive value of 95.5%. The SUNDROP authors concluded that telehealth screening can be safe, reliable, and cost-effective when coupled with committed ROP specialists to interpret images and perform live examinations when necessary.

A similar retrospective “real-world” study from an NICU in Montana reported good outcomes in the 137 infants evaluated, 13 of whom required transfer, and 9 of those transferred ultimately required laser treatment.^253^ Over the 4.5 years covered by their review, the authors noted no infants progressing to stage 4 or stage 5 ROP. The investigators followed the SUNDROP protocol for their screening schedule and ensured all infants were seen for a live diagnostic examination within 2 weeks of NICU discharge.

##### International

Vinekar et al. reported results from 36 rural NICUs in the southern Indian state of Karnataka starting in February 2011 through February 2015, covering remote screening of 7,106 infants as part of the Karnataka Internet Assisted Diagnosis of Retinopathy of Prematurity (KIDROP) program.^254^ The overall incidence of any ROP was 22.4% and treatment-requiring ROP was 3.6%. In this report, there was no comparison with criterion standard examination. The group's prior 2014 report examining 1,601 infants^255^ did compare nonphysician grading of images versus expert live examinations, but did not clearly report the results of this evaluation.

A national network for ROP screening was also developed and implemented in 11 NICUs in Chile.^256^ Images were taken by trained nonphysician operators using the RetCam Shuttle and were evaluated independently by two ROP experts. Of the 5,263 imaging sessions performed, 4,903 (93%) were considered good or excellent quality with evaluation of ROP possible in 98% of images. In this network, all screening and examinations were performed by telemedicine, with the exception of BIO examinations performed before treatment. Forty-two infants (4%) were referred for treatment and 98% agreement was found between the initial imaging and clinical examinations.

Lorenz et al. reported their 6-year experience with the wide-field remote screening in five NICUs in Germany.^257^ In this study, all 1,222 infants also received live examinations for comparison. The authors report that all 42 cases requiring treatment were successfully identified by telescreening with an acceptable positive predictive value of 82.4%.

In France, implementation of a telemedicine program for ROP screening resulted in an absolute 57.3% increase in the proportion of examinations completed in accordance with American Academy of Pediatrics guidelines, whereas the screening rates in the control group, which continued ROP screening using live examinations, remained unchanged.^258^ The average cost of examination in the telemedicine program was slightly more expensive (∼$22) than the standard procedure of transferring infants to a specialized center for examination by a specialist, but the authors projected that this cost would decrease as the number of examinations completed rose.^258^

Another French ROP screening program was conducted in Bordeaux between July 2009 and August 2015 and screened 419 infants using the RetCam 120.^259^ They found any ROP in 27.68% of infants. The authors felt that their exclusively telemedicine screening system was successful at identifying ROP, but did not report any data with regard to predictive values.

Skalet et al. performed a feasibility study on 26 babies in Lima, Peru.^260^ In this study, 95–97% of image sets were judged to be suitable for ROP grading.

Despite lack of full endorsement in current guidelines, remote ROP screening programs are being developed and implemented by many groups around the world.

#### Guidelines for Imagers

Although the use of nonphysician imagers is the foundation to the widespread use of telemedicine in ROP screening, it is imperative that imagers are appropriately trained and certified to ensure high-quality images. A team of at least two people is recommended for image acquisition: a certified retinal imager to capture images and NICU nurse to monitor the infant.^261^ Initial education of imagers in the e-ROP study consisted of general training on ROP, premature infants, and image acquisition including positioning infants and maintaining comfort. On-site instruction was provided by the camera manufacturer, including hands-on training with the camera and practice with a model eye. Imagers were also trained on image selection, data entry, and export of images.

Certification in the e-ROP study consisted of knowledge assessments and a practical examination that included submitting three bilateral image sets per protocol from infants. Images were evaluated by the reading center and feedback was provided with additional image sets submitted until sufficient quality was obtained. After certification, feedback was provided to sites monthly during calls and yearly at group meetings. The authors found that it was important for imagers to frequently image a varied patient population to maintain optimal skills. The e-ROP study demonstrated a 92% success rate for nonphysician imagers providing acceptable quality images.

In addition, the KIDROP program has developed a 90-day training that is available through an e-learning platform “WISE-ROP.”^®262^ Imagers read modules and complete quizzes to evaluate their progress. Video sessions and oral trainings are used to discuss the imagers' technique and hands-on sessions are scheduled with an assigned mentor.

A rigorous training and certification program is necessary for implementation of telemedicine in ROP screening to ensure high-quality images are consistently acquired.

#### Imaging Systems

One of the major considerations in any ROP telemedicine program is the choice of digital imaging system. Until 2016, most reports on ROP telehealth programs used the RetCam^®^ system.^218,245^ There are now several wide-field contact imaging systems on the market, although none have published clinical validation studies. The Visunex Panocam (Visunex Medical Systems, Inc., Fremont, CA) system has two cameras in its product line,^263^ a smaller portable system and a larger console system. The Phoenix ICON^®^ system, a contact wide-field cart-based system (Phoenix Technology Group, Pleasanton, CA), has also recently been introduced.^264^ The 3Nethra Neo^®^ (Forus Health, Bangalore, India), a 120° FOV, contact camera, has also recently been introduced.^262^

In a small pilot study of 128 premature infants from 35 NICUs, images acquired by both the Neo and RetCam were evaluated by two masked ROP specialists.^265^ The Neo was reported to have sensitivities of 97.4% and 99.3% and specificities of 81.1% and 75.6% for each grader, respectively. Since initial reporting, the study has been expanded to include 1,200 infants, but results are not yet published.

The Pictor^®^ (Volk Optical, Inc., Mentor, OH), a handheld noncontact fundus camera, has also been shown to be effective in screening for type 1 ROP and preplus and plus disease, despite its 45° FOV.^266,267^ The Pictor was found to have 100% sensitivity by both graders and 93% and 74% specificity by each grader, respectively, when compared with clinical examinations.^267^ At ∼$10,000, the Pictor may make implementation of telemedicine ROP screening programs more widely accessible.^267^

With the introduction of new cameras to the commercial market, investigators have found entry prices to be 40–50% of the recent past prices,^268^ making remote ROP screening systems more widely accessible.

#### Guidelines for Reading Centers

Although current guidelines recommend that graders for telemedicine ROP screening programs be experienced ophthalmologists who have experience in bedside examination as well as interpretation of digital images, several studies have examined the efficacy of the use of nonphysician graders.^237^ Nonphysician graders in the e-ROP study underwent a three-phase process including training, precertification, and final certification.^269^ Phase 1 of training included lectures that covered classification of ROP, the study and grading protocol, and current ROP treatments, interactive sessions with sample images, and a visit to an NICU to observe the imaging process.

To progress to phase 2, graders were required to pass a knowledge assessment. Phase 2 included grading of an average of 15 image sets along with review and discussion of the results compared with an expert consensus generated final result. Phase 3 included grading of 100 ROP training image sets with additional images added until 85% agreement was met. Final certification consisted of 15 image sets from the e-ROP pilot submission and was earned once 80% agreement was met. If this level of agreement was not achieved, retraining was performed for 1 week and the final certification with new images was repeated. This process was repeated until 80% agreement was met. After using this system, the authors reported a weighted kappa of 0.72 for intergrader agreement for RW-ROP as well as weighted kappas ranging from 0.57 to 0.94 for intragrader agreement for RW-ROP.

Despite the current guideline recommendations for physicians to evaluate digital images, there is inconsistent training on ROP and no standardized method of assessing competency among ophthalmology residency and pediatric ophthalmology and retina fellowship programs.^270^ The Global Education Network-ROP group has created a tele-education program for ROP to further the education of physicians evaluating ROP images. The program includes a pretest, ROP tutorial on classification and management, five training chapters that each emphasize a particular category of ROP, and a post-test. This education system has been studied in two separate populations: 31 ophthalmology residents among 5 residency programs in the United States and 1 residency program in Canada and 58 ophthalmology residents and fellows from 1 program in Mexico.^270,271^

Both studies found that the system was effective in improving diagnostic accuracy of ROP by ophthalmologists-in-training. Although this program has limitations, such as not tracking common errors made by trainees and not including examples of stage 4 or stage 5 ROP, the authors feel that improvements can be made and that this platform has potential to be used in a widespread manner to standardize evaluation of ROP images for both physician and nonphysician graders. Appropriate training of both physician and nonphysician graders is essential to ensure patient safety.

#### Automated Image Analysis

In concert with early reports of successful application of retinal photographic screening for ROP, interest in automated image analysis for image interpretation was also evident. Several earlier groups sought to determine whether the vascular tortuosity of plus disease could be segmented in an automated or semiautomated manner.^272,273^

Subsequently, several groups focused on integrated grading systems. Ataer-Cansizoglu et al., who are part of the i-ROP consortium, reported on their validation study in which 77 wide-angle images were graded by a computer algorithm “developed to extract tortuosity and dilation features from arteries and veins.”^274^ The algorithm grades were compared with a reference standard diagnosis generated by combining three independent expert image grades with the diagnosis rendered during a live BIO examination. The investigators found that their system was 95% accurate for the classification of preplus and plus disease, which compared favorably with the individual accuracy of the expert grades and was substantially higher than the mean accuracy of 31 nonexperts.

The i-ROP consortium also evaluated the methods physicians currently use when diagnosing ROP to further understand what may work best in an automated system. They found that ROP experts consider tortuosity of both arteries and veins and also consider areas outside the central retina when diagnosing plus disease, contrary to the International Classification of Retinopathy of Prematurity standards.^233^ They found that the performance of the i-ROP CBIA performed better than 9 of 11 experts in the study with 95% accuracy for diagnosis of plus disease when using a larger FOV than recommended and considering all vessels.^233^

Abbey et al. have also recently reported their validation of the ROPtool system.^275^ For this study, 335 fundus photographs were collaboratively assessed by a panel of three ROP experts to generate the criterion standard grade. Each quadrant was graded on a 5-point scale that incorporated tortuosity and dilatation. If any quadrant was graded as questionable or worse, then the image was classified as abnormal. The ROPtool system calculates the tortuosity of a single vessel within each quadrant and the value for the second most tortuous segment is defined as the tortuosity score for that eye. Dilation was also assessed, but this did not improve their model accuracy and was not presented.

The authors then examined multiple diagnostic set points through ROC. Optimizing sensitivity and including unreadable images as diseased, ROPtool had a sensitivity of 96% and specificity of 64%. The clinical utility of the proxy of tortuosity used as the criterion standard for this validation was not discussed.

More recently, deep learning, where CBIA systems have been trained to automatically recognize and evaluate images, has been used for ROP screening.^276,277^ Deep learning allows the system to continually learn and re-evaluate its process autonomously and consists of multiple layers of algorithms that data flow through to form neural networks.^277^ CNNs have to be trained through exposure to a large number and variety of pathological and normal images to then apply a series of filters to produce the desired output, which in this case would be diagnosis or classification of ROP.^277,278^

The i-ROP consortium has developed a deep learning algorithm, which has shown a high accuracy for identifying plus disease.^278^ The system was trained using a set of 5,511 retinal images that had been obtained as part of the i-ROP study and a reference standard diagnosis established by three trained graders and one expert clinical examiner. The system was able to diagnose plus disease on an independent set of 100 images with 93% sensitivity and 94% specificity and preplus disease or worse with 100% sensitivity and 94% specificity. The algorithm out-performed six of eight ROP experts and all prior computer-based imaging analysis systems in ROP without the need for manual segmentation.^278^

After the algorithm was trained to recognize plus disease, the authors also tested its ability to identify diagnostic categories and overall disease severity. After analysis of 4,861 images, they found that the system could accurately detect clinically significant ROP with 94% sensitivity and a 99.7% negative predictive value based on posterior pole fundus photographs alone.^276^

Wang et al. also developed two deep neural networks, Id-Net and Gr-Net, which were, respectively, designed for the identification and grading of ROP.^279^ Id-Net achieved a sensitivity of 96.62% and specificity of 99.32% for identification of any ROP, and Gr-Net achieved 88.46% sensitivity and 92.31% specificity for grading of ROP severity, which was comparable with three expert graders.

Zhang et al. have also evaluated three general-purposed deep neural networks (AlexNet, GoogLeNet, and VGG16) using a transfer learning workflow with 17,801 images to identify ROP.^280^ They found that VGG16 achieved the best performance on a test set of 1,742 images and found that this performance was comparable with that of five pediatric ophthalmologists.

The use of CBIA systems in ROP screening could help improve the accuracy and consistency of diagnosis of ROP.

#### Safety of Retinal Imaging in ROP

Although many remain skeptical of the safety of remote ROP imaging and grading of images,^238^ several studies have reported low frequencies of AEs associated with retinal imaging. In the e-ROP study, one-third of AEs were reported to have probably or definitely been related to BIO (4 AEs) or contact imaging (18 AEs).^281^ Based on the low frequency of AEs (65 AEs reported >4,238 visits) and serious AEs (none) reported in the e-ROP study, the authors considered both BIO and imaging to be safe methods of ROP screening.^281^

Prakalapakorn et al., who have examined the use of the Pictor noncontact fundus camera, also found that safety events (clinically significant bradycardia, tachycardia, oxygen desaturation, or apnea) occurred after 5.8% of clinical examinations and after 0.8% of imaging sessions.^282^ Because the noncontact camera did not require a use of a lid speculum or contact with the cornea, the authors felt that the process was less stressful for infants.

Despite the survey finding in which only 30% of NICU directors felt that telemedicine for ROP screening was safe, studies evaluating AEs surrounding the use of ROP imaging have so far found low incidences of AEs.

#### Costs of Remote ROP Screening

A major barrier to implementation of telemedicine in general is the high startup cost. Within ocular telehealth, the retinal cameras needed for imaging premature infants are costlier than the nonmydriatic devices used to image adults with diabetes, but costs are decreasing with the release of new cameras, such as the Neo, and expansion to nontraditional cameras, such as the Pictor.

Several cost-effectiveness analyses have been published exploring different scenarios for ROP screening. Jackson et al. performed a cost–utility analysis of telemedicine and standard ophthalmoscopy compared with no treatment from a third-party perspective.^283^ This group found that the cost per QALY gained was $3,193 for telehealth screening compared with $5,617 with standard ophthalmoscopy.

Varying several aspects within the simulation generated wide variations from their base case (up to $18,989 per QALY gained for telehealth and $27,215 for ophthalmoscopy), but the interventions remained below the previously described threshold of a highly cost-effective intervention of $50,000/QALY. Because the perspective chosen for this analysis (third-party payer) does not include the costs of acquiring the retina cameras and telehealth connectivity, the results are valuable in convincing policy makers and insurers of the value of the intervention, but do not necessarily speak of the viability of establishing a telehealth program for hospitals.

Castillo-Riquelme et al. likewise performed a cost-effectiveness of retinal photographs screening for ROP in the United Kingdom.^284^ This simulation study compared five different screening strategies and used a health system perspective. The investigators estimated that the current methods cost GBP 321 to screen one infant and that if a specialist nurse were to travel among NICUs to capture and interpret images, this would be substantially less expensive (GBP 172 per infant or GBP 201 if the images were transmitted for ophthalmologist review).

Other methods explored in their simulation would be more expensive: use of a standard camera with NICU nurses acquiring and interpreting the images (GBP 371) or transmitting the images for ophthalmologist review (GBP 390). Throughout the sensitivity analysis, the least expensive method was largely unchanged, unless the cost of the visiting nurse was almost at the extreme high end of the sensitivity range or the specificity of nurse interpretation was 40% or less (99% was used in the base case). Of note, if the sensitivity dipped slightly <90%, the standard examination strategy was noted to be “cost-effective.” The authors suggest that development of a portable imaging solution could dramatically change the cost-effectiveness landscape. The results may be difficult to apply to other settings without a national health system.

Makkar et al. noted that implementing telemedicine examinations for ROP in a level II NICU reduced costs associated with transport, decreased the length of hospitalization, and decreased the use of higher levels of care than needed.^285^ They also noted that the current telemedicine reimbursement rate for digital retinal examinations does not cover the cost of required effort (∼1 h of total processing time for each infant imaged).

### Conclusions

As the preceding discussion illustrates, the ocular telehealth paradigm for ROP is different from remote screening for DR as discussed in the body of these guidelines. The population at risk is hospitalized low-birth weight premature infants, so the technical aspects of image acquisition need to account for the NICU environment and the anatomy of the neonatal eye. For ROP, the burden of screening largely falls on the providers and health systems rather than on patients to present for opportunistic screening. Perhaps the most critical difference is the time course of vision loss and prognosis for eyes in which the ROP diagnosis is not made in a timely manner. Unlike in DR, very high sensitivities for vision-threatening disease must be achieved because the risk of near-term potentially permanent vision loss is unacceptably high.

In addition to the challenges posed by ROP for effective ocular telehealth, there are opportunities that are unique to ROP. Because the screenings are done in a controlled environment, universal coverage of screening should be far easier to achieve than in DR, and technical issues with equipment may be easier to deal with in the NICU environment with access to hospital IT and biomedical engineering departments. DR telehealth screening programs sometimes are met with resistance from primary eye care providers as the programs can be seen as a threat to patient volumes and revenue streams. ROP screenings, in contrast, are often a challenge for hospitals to find appropriate coverage and could provide more convenience to treating providers rather than “competition.”

Finally, there may be medical–legal benefits to photo-documentation of ROP examination findings, particularly if there are automated aids to image classification or decision support within the grading software.

Any ROP screening implementation using telehealth should follow the screening recommendations of the major societies of the region.^235^ Retinal images must be of sufficient quality to allow a grader to make an accurate determination of the ROP status. Different groups have used different diagnostic set points as already discussed, so any program must validate to their predetermined level of disease severity, analogous to the recommendations made in the body of this document for DR.

The majority of research to date has used contact wide-field imaging, but research is ongoing to determine the value of noncontact posterior pole imaging to detect plus disease to screen for RW-ROP.^286^ Because the volume of babies requiring imaging in a given center may be lower than what is seen in DR screening, deliberate efforts should be taken so that imagers maintain skills. Further technical guidance is provided in a 2015 Joint Technical Report of the American Academy of Pediatrics, the American Academy of Ophthalmology, and the American Association of Certified Orthoptists.^229^

### Disclosure Statement

J.P.C. receives grant support from Genentech, and is listed on a preliminary patent application related to deep learning technology for ROP screening.

### Funding Information

C.J.B. was supported by the National Institute of General Medical Sciences of the National Institutes of Health under Award Number P20GM103644. The content is solely the responsibility of the authors and does not necessarily represent the official views of the National Institutes of Health. S.D. was supported in part by the Elliot W. Shipman Professorship Fund. J.P.C. was supported by National Institutes of Health grants R01EY19474, P30EY10572, and K12EY27720 (Bethesda, MD), National Science Foundation grant SCH-1622679 (Arlington, VA), and unrestricted departmental funding and a Career Development Award (JPC) from Research to Prevent Blindness (New York, NY).

### Appendix A11. Telemedicine for Age-Related Macular Degeneration

Christopher J. Brady, MD, MHS,^1^ and Seema Garg, MD, PhD^2^

^1^Division of Ophthalmology, Department of Surgery, Larner College of Medicine, University of Vermont Medical Center, Burlington, Vermont.

^2^Department of Ophthalmology, University of North Carolina, School of Medicine, Chapel Hill, North Carolina.

### Introduction

AMD is the leading cause of vision loss in the United States.^287^ As such, the disease presents an appropriate target for ocular telehealth interventions. Unlike DR, there is no consensus about the utility of population screening for AMD.^288^ Some groups have found value by adding screening for AMD to existing DR screening programs,^289,290^ but others have not found screening programs for AMD to be cost-effective.^291^ Several groups have investigated the feasibility and validity of telehealth programs for AMD. Owing to the uncertainty of the role of screening for AMD, several groups have explored other telehealth paradigms for this disease.

### Clinical Feasibility

Although the official gold standard for DR diagnosis is seven-field, ETDRS stereoscopic fundus photography, the benchmark for the diagnosis of AMD remains a combination of clinical examination, and fluorescein angiography and OCT. Therefore, several groups have sought to validate the use of fundus photographs for the diagnosis of AMD through the detection of characteristic lesions of AMD (drusen, hyperpigmentation, CNVM, and geographic atrophy [GA]). Specifically as it relates to the presence of CNVM, a retrospective analysis of stereoscopic images of 127 fellow eyes from the Macular Photocoagulation Study correctly identified all 30 eyes that developed CNVM as defined by the fluorescein angiogram.^292^

To determine the accuracy of diagnosing AMD with monoscopic images alone, Scholl et al. at Moorfields Eye Hospital compared digitized color, mydriatic, monoscopic images with stereoscopic 35 mm slides and found an agreement of 83–93% for the presence or absence of intermediate drusen depending on the macular subfield examined.^293^ Furthermore, they found agreement of 94–96% for GA and 94–98% for CNVM.

Pirbhai et al. conducted a prospective comparison of mydriatic monoscopic color fundus photographs with conventional clinical evaluation and fluorescein angiography.^294^ In this study, the diagnoses rendered on the basis of the monoscopic images were 89.2% sensitive and 85.7% specific. Clinical recommendations based on the monoscopic images corresponded to the gold standard clinical examination 80.3% of the time. The kappa statistic is frequently used to test inter-rater reliability and can range from −1.0 to 1.0. In this study, the kappa was 0.59, which the authors concluded was evidence of good agreement.^294^

In another study, Duchin et al.^295^ compared nonmydriatic fundus images with a conventional clinical dilated fundus examination with a retina specialist. In 94 eyes of 47 patients,^295^ they found sensitivity for referable AMD (Age-Related Eye Disease Study [AREDS] grading level 3 or greater) to be 84–88% and specificity of 81% between their two expert graders. Of note, the authors used their existing telemedicine infrastructure for DR screening.

### Clinical Experience

To expand upon feasibility studies, several groups have implemented ocular telehealth programs for AMD. In a randomized controlled trial, participants referred for possible or established neovascular AMD were randomly assigned to either conventional clinical examination or image acquisition and remote interpretation at an ocular telehealth site.^296^ Data collected included best-corrected visual acuity, intraocular pressure, color fundus photographs (mydriatic status not specified in publication, but clarified as dilated by senior article author, Dr. Thomas Sheidow, pers. comm., February 9, 2018) and OCT.

These data were transmitted to retina specialists at a tertiary referral site. They found no delay in presentation for care in the telescreening group, but did note increased interval between detection and reinitiation of therapy in participants with established AMD. They did not detect any adverse outcomes in terms of visual acuity attributable to this delay in this small study.

Another *in situ* study was performed by De Bats et al. in Lyon, France.^297^ In this study, 1,022 individuals were screened for known presence of AMD and absence of comorbidity that would preclude AMD management, of whom 683 were eligible and interested in participating. Nonmydriatic color photographs were then taken at two community health examination centers and then transmitted for grading by an ophthalmologist. Images were gradable in 80% of the 1,363 images acquired, and AMD was diagnosed in 178 eyes. There was no gold standard assessment of participants in this study.

As in DR, ROP, and other retinal conditions, there is growing interest in the use of AI systems for image processing and interpretation.^298^ Such systems may allow for more rapid/instantaneous grading of images with accuracy similar to expert human grading. Investigators have used a variety of public and private data sets including the Singapore Integrated Diabetic Retinopathy Screening Programme^136^ and the AREDS^299,300^ to train deep learning algorithms to identify features of AMD on color fundus photographs. Other groups have likewise used deep learning approaches to identify AMD on OCT images.^301,302^ At the time of this writing, no AI system is FDA approved for AMD.

### Remote Monitoring

Several groups have looked to other telehealth paradigms beyond store-and-forward remote screening/detection, such as remote monitoring. Andonegui et al. sought to determine whether ancillary testing performed without a live examination could allow clinicians to reach a similar assessment and plan to that diagnostic decisions based on a live examination.^303^ In this study, 201 participants with exudative AMD who had received a minimum of three prior ranibizumab injections initially had a live examination with spectral domain OCT (SD-OCT), fundus photography, and visual acuity measurements. Antivascular endothelial growth factor (VEGF) retreatment decisions were made based on this live examination and recorded.

At least 4 weeks later, the ancillary data were anonymized and randomly distributed to the same two retina physicians who had seen them previously. A retreatment decision was then rendered and recorded based on this “remotely acquired” clinical data, simulating a telehealth encounter. The same treatment decision was reached in 90% of cases, with 8% of patients receiving “false-positive” (i.e., the remote decision was to retreat, but the live decision was to defer), and 1% receiving a “false-negative” (i.e., the remote decision was to defer, but the live decision was to treat).

Moving further away from remote image acquisition and transmission, Azzolini et al. sought to determine whether an e-health decision support tool could help general ophthalmologists follow AMD patients without referral.^304^ General ophthalmologists could enter in patient age, visual acuity, Amsler grid results, presence of macular hemorrhage, and fellow eye status. A risk score for active exudation is calculated, and the general provider can directly schedule with a retinal provider in instances of high risk. A comparison of consecutive patients undergoing usual care was also established.

During the study period, 360 patients with known AMD were examined within the network. Of these, 310 were judged high risk of disease progression, and referred for a live examination. Of these, 276 received intravitreal anti-VEGF therapy. There was less of a delay before initiating therapy in the “network” as compared with the usual care patients, and all providers judged the system to be “good” or “very good.” The validation of risk score is listed as “unpublished data” and the 50 patients with low-risk scores were not examined in this study.

Another approach at remote monitoring using a consumer device was explored in the AREDS 2 study.^305^ In this study, participants with nonexudative AMD at high risk for developing choroidal neovascularization (CNV) were randomized to either use the ForeseeHome device daily at home or to standard-of-care symptom monitoring. The device tests macular visual field using hyperacuity techniques, and sent an alert to investigators if a substantial change was noted. During a prespecified interim analysis, a statistically significant smaller decline in visual acuity was noted at the time of diagnosis of active CNV in the ForseeHome group as compared with standard-of-care monitoring. For this reason, early termination for efficacy was recommended. After FDA approval of the device, a cost-effectiveness analysis from a federal government perspective found that home telemonitoring of patients at high risk for CNV was cost-effective compared with biannual in-person examination.^306^

### Conclusions

Across a range of studies, then, numerous different ocular telehealth strategies have been tested for AMD. Because of the lack of well-defined high-risk population, merely extending existing DR screening pathways to screen for AMD is not currently in use, nor recommended. Ocular telehealth for AMD is likely to require expansion of the remote screening tool kit of a network-connected nonmydriatic fundus camera to include technologies such as OCT and possibly OCT angiography. As new strategies are tested in high-quality studies, and as the population ages, and the burden of AMD increases, there will likely be opportunities for remote monitoring, either through teleconsultation with general medical providers or optometric or general ophthalmologic providers, or through consumer facing home monitoring. Indeed, finding solutions to the challenges of remote detection and management of AMD may allow for the generalization of ocular telehealth methods to a number of different conditions, and may help usher the field away from a disease-specific paradigm.

### Disclosure Statement

No competing financial interests exist.

### Funding Information

C.J.B. was supported by the National Institute of General Medical Sciences of the National Institutes of Health under Award Number P20GM103644. The content is solely the responsibility of the authors and does not necessarily represent the official views of the National Institutes of Health.
